# Genetic and transcriptional variations in *NRAMP*-*2* and *OPAQUE1* genes are associated with salt stress response in wheat

**DOI:** 10.1007/s00122-018-3220-5

**Published:** 2018-11-03

**Authors:** Benedict C. Oyiga, Francis C. Ogbonnaya, Ram C. Sharma, Michael Baum, Jens Léon, Agim Ballvora

**Affiliations:** 1 INRES-Pflanzenzuchtung, Rheinische Friedrich-Wilhelms-Universitat, Bonn, Germany; 2 Center for Development Research (ZEF), Rheinische Friedrich-Wilhelms-Universitat, Bonn, Germany; 3 Grains Research and Development Corporation, Kingston, ACT 2604 Australia; 4 International Center for Agricultural Research in the Dry Areas (ICARDA), Tashkent, Uzbekistan; 5 International Centre for Agricultural Research in the Dry Areas (ICARDA), Al Irfane, 10112 Rabat, Morocco

## Abstract

**Key message:**

SNP alleles on chromosomes 4BL and 6AL are associated with sensitivity to salt tolerance in wheat and upon validation can be exploited in the development of salt-tolerant wheat varieties.

**Abstract:**

The dissection of the genetic and molecular components of salt stress response offers strong opportunities toward understanding and improving salt tolerance in crops. In this study, GWAS was employed to identify a total of 106 SNP loci (*R*^2^ = 0.12–63.44%) linked to salt stress response in wheat using leaf chlorophyll fluorescence, grain quality and shoot ionic (Na^+^ and K^+^ ions) attributes. Among them, 14 SNP loci individually conferred pleiotropic effects on multiple independent salinity tolerance traits including loci at 99.04 cM (*R*^2^ ≥ 14.7%) and 68.45 cM (*R*^2^ ≥ 4.10%) on chromosomes 6AL and 4BL, respectively, that influenced *shoot Na*^+^-uptake, *shoot K*^+^*/Na*^+^ ratio, and specific energy fluxes for absorption (*ABS*/*RC*) and dissipation (*DIo*/*RC*). Analysis of the open reading frame (ORF) containing the SNP markers revealed that they are orthologous to genes involved in photosynthesis and plant stress (salt) response. Further transcript abundance and qRT-PCR analyses indicated that the genes are mostly up-regulated in salt-tolerant and down-regulated in salt-sensitive wheat genotypes including *NRAMP*-*2* and *OPAQUE1* genes on 4BL and 6AL, respectively. Both genes showed highest differential expression between contrasting genotypes when expressions of all the genes within their genetic intervals were analyzed. Possible cis-acting regulatory elements and coding sequence variation that may be involved in salt stress response were also identified in both genes. This study identified genetic and molecular components of salt stress response that are associated with *Na*^+^-*uptake*, *shoot Na*^+^*/K*^+^ ratio, *ABS*/*RC*, *DIo*/*RC*, and grain quality traits and upon functional validation would facilitate the development of gene-specific markers that could be deployed to improve salinity tolerance in wheat.

**Electronic supplementary material:**

The online version of this article (10.1007/s00122-018-3220-5) contains supplementary material, which is available to authorized users.

## Introduction

Wheat is one of the world’s most important strategic food crops, with annual production of about 736 million metric tons (MMT) (FAO 2015). Due to the continuous decline in global production of major staple food crops since 1980 caused by global warming (Lobell et al. 2011), there is a need to increase wheat production amidst the projected increase in the world’s population (Jaggard et al. 2010) and the threat posed by continuous salinization of arable lands and ongoing climatic change. Over 6% of the world’s total land area is affected by salinity (FAO 2010). This is expected to increase in the coming years (Munns and Tester 2008), resulting in predicted US$12 billion annual losses in agricultural production (Qadir et al. 2008; Flowers et al. 2010). Soil salinization is an important land degradation problem in both dry and irrigated areas. Salinization can be reduced by leaching soluble salts out of the soil with excess irrigation water (Gupta and Goyal 2017), and/or high levels of soil salinity can be tolerated if salt-tolerant crops are grown (Ashraf et al. 2012). High salt concentration in soils reduces plant growth (Munns and Tester 2008; Zhang et al. 2010a) by limiting leaf growth and expansion (Cramer 2002). Salt-tolerant and salt-sensitive crops respond differently to salinity stress. The former tend to exclude excess salts (such as Na^+^) from the roots as well as compartmentalize the excess Na^+^ in leaf vacuoles to avoid damaging the photosynthetic machinery.

Salt stress leads to ionic imbalance and hyperosmotic stress in plants (Flowers 2004), causing severe damage to photosynthetic apparatus (Parida and Das 2005; Yamane et al. 2008) and reduction in seedling growth and survival rate (Lutts et al. 1995) which results in poor seed set and low crop yield (Asch et al. 2000; Atkinson and Urwin 2012; Nayidu et al. 2013). Grain quality is also adversely affected by salinity (Rao et al. 2013) due to the indirect effects of Na^+^ toxicity on the photosynthetic and biosynthetic processes within the chloroplasts. Under high salinity, the soil available K^+^ and Na^+^ compete for uptake in plant roots, and the degree to which one ion is preferred over the other determines adaptation and tolerance to salt stress. Zhang et al. (2010a) indicated that low K^+^- and high Na^+^-uptake are often correlated with each other. Thus, the identification of salt-tolerant genotypes possessing attributes such as reduced uptake of Na^+^, increased preference of K^+^ over Na^+^ in the root during uptake and translocation to shoots, increased tissue tolerance and production of enzymes, hormones, antioxidants, etc., is of great interest to wheat breeders. To meet future grain demands, breeders are required to develop new resource-efficient and salt-tolerant wheat cultivars by combining large-scale plant phenotyping with genome sequencing in forward genetics. Over the years, the traditional phenotyping methods have been used to evaluate crops agronomic and yield performance under salinity stress. However, these methods are labor-intensive, time-consuming and are considered to be one of the major bottlenecks in exploiting genetic information for genomic analysis (Rahaman et al. 2015) and advancement of yield improvement (Furbank and Tester 2011; McCouch et al. 2013). The evaluation of large populations in breeding programs has necessitated the search for traits linked to salinity tolerance that are economical, quick, and easy to measure; to facilitate the identification and mapping of salt tolerance (ST) QTL. The use of chlorophyll fluorescence (HTP_chlF_) techniques allows for non-invasive plant analyses and quick detection of stress or damage in the photosynthetic apparatus (Glynn et al. 2003; Chaerle et al. 2007). In turn, the potential in understanding common genetic factors regulating leaf photosynthetic activities, *shoot K*^+^ and *shoot Na*^+^ accumulation and grain quality in wheat would be very useful to breeders in bridging the gene to productivity/grain quality gap under salt-affected agro-ecologies.

Progress is being made in the discovery of quantitative trait loci (QTL) linked to ST genes involved in cell protection, transporter and oxidoreductase activities and regulatory elements which enhance the plant response to salt stress (James et al. 2006; Byrt et al. 2007; Huang et al. 2009; Zhang et al. 2012; Li et al. 2013, 2014a; Shi et al. 2014). The variable expressions of these genes are essential components of plant salt stress response and adaptation mechanisms and may be genotype specific and/or transcriptionally regulated by cis-regulatory elements (CREs). Several studies have revealed the association of DNA polymorphisms with ST in wheat (Genc et al. 2010; Xu et al. 2013; Masoudi et al. 2015; Turki et al. 2015), but are yet to be elucidated due to lack of functional and molecular validation. The understanding of how potential molecular cues relate to salt stress response remains a major challenge in molecular biology, as transcriptional mechanisms contribute to the regulation of nearly all cellular processes (Li et al. 2006; von Korff et al. 2009). Such understanding would facilitate exploiting them in genomic research and breeding programs to improve wheat salt stress tolerance and productivity. However, strengthening the connection between genotype and phenotype is a major challenge facing by plant molecular breeders (Pauli et al. 2016). The advances in next-generation DNA sequencing (NGS) have made identification of QTL/genes associated with complex agronomic traits possible. Genome-wide association studies (GWAS) provide dense genome coverage and allow for accurate identification of putative QTL in unstructured populations (Lorenz et al. 2011). It has been routinely used to identify the genetic basis of simple and complexly inherited agronomic and adaptive traits (Jighly et al. 2015; McCouch et al. 2016; Sun et al. 2017).

In this study, we used noninvasive high-throughput FluorPen FP100 to assess the leaf photosynthetic activities of a wheat diversity panel under saline and non-saline stress conditions. In addition, *shoot K*^+^ and *Na*^+^ accumulation and grain quality (GQ) under salt stress conditions were also assessed. The objectives of this study were to: (i) identify HTP_chlF_ traits that may serve as possible surrogates for screening salt stress response in wheat, (ii) identify QTL linked to ST using the HTP_chlF_, shoot ionic and GQ traits and (iii) annotate functionally the associated SNP loci to uncover the putative genes involved and gain insights into their salt response mechanisms by in silico protein, expressions, promoter and expressed sequence analyses. To our knowledge, this is the first study that simultaneously evaluated and analyzed the genetic architectures of salt stress response in a wheat diversity panel using three independent parameters: leaf chlorophyll fluorescence, *shoot Na*^+^*/K*^+^ accumulation and grain quality.

## Materials and methods

### Plant material and growth conditions

The 150 wheat genotype diversity panels including the hydroponic screening systems used in this study have been described previously in Oyiga et al. (2016). Briefly, the germplasm was grown in three replicated hydroponic systems in the greenhouse between September and December 2016 under non-saline (control) and 150 mM NaCl (13.24–14.71 dS m^−1^) saline conditions. The experiment was repeated two times. Salt stress was initiated 3 days after planting by addition of 50 mM NaCl incrementally for 3 days. After this time, the full 150 mM NaCl treatment was sustained for 24 days until harvest.

### Leaf chlorophyll a fluorescence

At day 24 of salt stress treatment, three readings on the HTP_chlF_ traits were taken at three positions on the blade of the third leaf (c. 5 cm from the stem, toward the centre, and c. 5 cm from the tip) using the FluorPen FP100 (Photon Systems Instruments, Brno, Czech Republic). Readings were taken on the light-adapted leaves of six plants per genotype. In total, 18 data points per genotype were used for analyses. Light intensity reaching the leaves during the measurement was set at 3000 mol (photons) m^−2^ s^−1^, which was sufficient to generate maximal fluorescence. The HTP_chlF_ parameters evaluated (Strasser et al. 2004) are described in Table 1.

**Table 1:** List of measured leaf chlorophyll a fluorescence parameters measured

Traits	Formula explanation	Description
*Extracted and technical fluorescence*		
*F*_v_	*F*_v_ = *F*_m_ − *F*_o_	Maximal variable fluorescence
*F*_m_/*F*_o_		
*F*_v_/*F*_o_	(*F*_m_ − *F*_o_)/*F*_o_	Efficiency of the water-splitting complex on the donor side of PSII
*F*_v_/F_m_		Maximum quantum yield of PSII within light-adapted
*Specific fluxes or specific activities* (*per Q*_*A*_-*reducing PSII reaction center*—*RC)*		
ABS/RC	ABS/RC = Mo × (1/Vj) × (1/Phi Po)	Effective antenna size of an active reaction center (RC). Total number of photons absorbed by Chl molecules of all RC divided by the total number of active RCs
TRo/RC	TRo/RC = Mo × (1/Vj)	Maximal trapping rate of PSII. Maximal rate by which an excitation is trapped by the RC resulting in the reduction of QA to QA−
ETo/RC	ETo/RC = Mo × (1/Vj) × Psi o	Electron transport in an active RC. Re-oxidation of reduced QA via electron transport in an active RC. It reflects the activity of only the active RCs
DIo/RC	DIo/RC = (ABS/RC) − (TRo/RC)	Effective dissipation in an active RC. Ratio of the total dissipation of un-trapped excitation energy from all RCs with respect to the number of active RCs
*Quantum efficiencies or flux ratios*		
PI_(ABS)_		Performance index on absorption basis where
Psi_0	1 − V_J_	probability that a trapped exciton moves an electron into the electron transport chain beyond QA
Phi_Po	Phi_P0 = 1 − (*F*_0_/*F*_M_) (or *F*_V_/*F*_M_)	Yield of primary photochemistry

### Shoot K^+^ and Na^+^ accumulation during salt stress

The amount of Na^+^ and K^+^ (in g/100 g sample DW) accumulated in the shoot (leaf parts plus stem) after 24 days of salt treatment was measured using atomic absorption spectrophotometer (type 2380; PerkinElmer, Wellesley, MA, USA), as described in Oyiga et al. (2016), followed by the calculation of their corresponding K^+^ to Na^+^ ratios.

### Determination of the grain quality (GQ)

The GQ traits including grain protein content (GPC), grain starch content (GSC), grain neutral detergent fiber (NDF) and grain crude fiber (CFC) of the 150 wheat diversity panel were also measured from grains harvested from three replicated field trials in Karshi, Uzbekistan, under high-saline (9.24–17.58 ds/m) and low-saline (2.40–6.34 ds/m) field conditions. The GQ analysis was performed using a DA 7250 NIR analyzer (PerkinElmer, Wellesley, MA, USA).

### Statistical analyses of the phenotype data

Due to the positioning (row and column) effects in the experiments, all the traits evaluated were analyzed following the restricted maximum likelihood (REML) procedure as implemented in GENSTAT 16. The plant position effects were accounted for by including “Replication/Row*Column”—rows crossed with columns nested within replication as sources of random effects in REML (Payne et al. 2015). Statistically significant differences were ascertained with the Wald’s test. Variance components due to genotypic ($\sigma_g^2$) and environmental ($\sigma_e^2$) effects were determined [by considering genotypes as random effect and, by taking into account the genotype by salt-treatment interaction (Piepho et al. 2008)] and were used to estimate the traits broad-sense heritability (*h*^2^) as implemented in GENSTAT 16 for REML (O’Neill 2010) as: $h^2 = \sigma_g^2/(\sigma_g^2 + \sigma_{gT}^2/T + \sigma_R^2/r)$, where $\sigma_{gT}^2$ is genotype × salt-treatment interactions variance, T is the number of number of treatments, $\sigma_R^2$ is the residual *variance and* r is the number of replications in each treatment. Best linear unbiased predictors (BLUPs) were used to calculate the salt tolerance indices (STIs) of all the traits, as described in Oyiga et al. (2016). The STI estimates were included in the GWAS model as phenotypes for the localization of QTL associated with salt stress response in the diversity wheat panel. Correlation coefficients (*r*) for each pair of STI trait were also obtained using the SPSS 16.0 program for windows.

### Genetic analysis of the 150 wheat diversity panel

Detailed information of SNP genotyping, population structure (PS) and linkage disequilibrium (LD) analyses of the diversity panel have been described in Oyiga et al. (2018). A subset of 18,085 polymorphic SNP markers remaining after data cleaning (missing values > 5% and minor allele frequency, MAF < 5%) were used for GWAS analysis which was performed with two software programs: TASSEL 5.2.13 standalone version (Bradbury et al. 2007) and SAS (SAS Institute Inc., Cary, NC). Only the congruent SNP loci identified by both programs were reported after employing the mixed linear model (MLM) adjusted for PS (*Q* matrix, as the fixed covariate) and kinship (K-matrix, as the random effect) matrixes. The cutoffs for accepting significant marker–trait associations (MTA) were calculated according to Long et al. (2013): -log10 (α/#tests), where *α* = 0.05 and #tests = the number of effective tests calculated as the total genome coverage divided by genome linkage disequilibrium (LD). Based on LD of the population, all SNP loci detected at genetic intervals defined by the genome LD of 10 cM for A-genome, 11 cM for B-genome and 14 cM for D-genome (Oyiga et al. 2018) were considered to be in high LD (Breseghello and Sorrells 2006; Pasam and Sharma 2014) and were grouped in one SNP cluster. Thereafter, the principal-coordinate analyses (PCoA) based on all the detected SNP loci were performed in GenAlEx 6.5 (Peakall and Smouse 2012) using the salt-tolerant and salt-sensitive wheat genotypes that were identified in the diversity panel (Oyiga et al. 2016), to check their genetic diversity for salt stress tolerance.

### In silico identification and ontological analysis of the detected SNP loci

Based on SNP marker sequence information, candidate genes containing associated SNP markers were identified using NCBI BLAST (www.ncbi.nlm.nih.gov/BLAST), as previously described in Oyiga et al. (2018).

### Gene expression analyses of candidate genes harboring the associated SNP loci

The third leaves harvested from 4-week-old *Altay2000* and *Bobur* after 2 h, 11 days and 24 days under saline (100 mM NaCl) and non-saline conditions were used for expression analyses of the candidate genes. *Altay2000* and *Bobur* are among the four wheat genotypes—*Altay2000* and *UZ*-*11CWA*-*8* (salt-tolerant) and *UZ*-*11CWA*-*24* and *Bobur* (salt-sensitive), which showed consistent and contrasting response to salinity stress across three different developmental growth stages identified in the GWAS panel assessment (Oyiga et al. 2016). The expression level of the candidate genes after 24 days of salt stress was included because it coincided with the time period at which the GWAS phenotypic data for both HTP_chlF_ and *shoot K*^+^- and *Na*^+^-accumulation were collected. The blades of the leaf samples from five plants per genotype were pooled and analyzed using the quantitative next-generation sequencing (NGS) based on the massive analysis of cDNA ends (MACE) transcriptome profiling.

### RNA isolation and RT-qPCR of the associated candidate genes

Leaf samples from the third leaf of three plants of *Altay2000* and *UZ*-*11CWA*-*8* (salt-tolerant), and *UZ*-*11CWA*-*24* and *Bobur* (salt-sensitive) wheat genotypes were collected after 30 days in both saline (100 mM NaCl) and non-saline conditions, flash frozen in liquid nitrogen and preserved at − 80 °C until use. Total RNA was extracted from the harvested leaves using the E.Z.N.A. Plant RNA Kit (Omega Bio-Tek, Norcross, GA, USA), followed by DNA digestion with the Omega Bio-Tek DNA Digestion kit. The qualities of the extracted RNA samples were verified with electrophoresis (1.5% agarose gel) and quantified spectrophotometrically using NanoDrop 2000c (PEQLAB *Biotechnologie GmbH*, 6404 Polling).

The cDNA synthesis was performed with first-strand cDNA Synthesis kit—Cat.#K1632 (ThermoFisher Scientific, Massachusetts, USA). Primer pairs (Table 2) were designed for *OPAQUE1* (*myosin*-*J heavy chain*) on 6AL, *ABC transporter F family member* 3 (*TaABCF3*) on 6AL and *NAD(P)H*-*quinone oxidoreductase subunit L* (*NAD(P)H*) on 5AL using the online Primer3 program (http://primer3.wi.mit.edu/). Gene amplification via RT-qPCR was carried out in biological triplicates of 20 μl final volume reactions containing 3 μl of cDNA template, 0.3 μl of each gene-specific primer, 10 μl of DyNAmo ColorFlash SYBR Green 2X-master mix with ROX- Cat.#F456L (ThermoFisher Scientific, Massachusetts, USA) and 6.4 μl RNase-free water, with the following cycling conditions: pre-denaturation 95 °C/7 min and then for 40 cycle: 95 °C/10 s, 60 °C/30 s, 72 °C/30 s. Gene amplification and fluorescence data acquisition were conducted on SDS-7500 Sequence Detection System (Applied Biosystems, USA) using cDNA obtained from the stressed and non-stressed salt-tolerant and salt-sensitive plants. The expressed gene transcript amounts were normalized with *TaEf*-*1a* and *TaEf*-*1b* (Unigene accession: *Ta659*; Table 2), following the Livak and Schmittgen (2001) method.

**Table 2:** RT-PCR primer pairs used for the endogenous control gene and amplification of selected wheat transcripts

Gene	Chr.	Forward primer (5′–3′)	Reverse primer (5′–3′)	Size (bp)
*Target candidate genes*				
*OPAQUE1*	6AL	GCCCAACGCCAGCAAAATA	GGATTCAAAAGCACGCCAGT	175
*TaABCF3*	6AL	ATTCCCAACCCCAGATGAC	ACTGTTCCCGATGTTGGTTG	210
*NAD(P)H*	5AS	GGATGAGGCAGAGGTGGTT	GCGGGTATCTGTCCTTGAAC	195
*Internal control genes*				
*TaEf*-*1a*	–	CTGGTGTCATCAAGCCTGGT	TCCTTCACGGCAACATTC	151
*TaEf*-*1a*	–	CAGATTGGCAACGGCTACG	CGGACAGCAAAACGACCAAG	227

Details of the primers used for the gene amplification and their corresponding product size. ***OPAQUE1***, *myosin*-*J heavy chain*; **TaABCF3,** ABC transporter F family member 3 and ***NAD(P)H***, NAD(P)H-quinone oxidoreductase subunit L (chloroplastic)

In many cases, the associations identified in GWAS may provide the initial landmarks for the identification of the candidate genes underpinning genetic variants and do not represent the causal effect but rather one in LD; the scaffolds corresponding to the genetic intervals (in LD) of the pleiotropic SNP loci detected at 68.45 cM and 90.04 cM on 4BL and 6AL chromosomal regions, respectively, were identified and analyzed using the JBrowse (Skinner et al. 2009) in the International Wheat Genome Sequencing Consortium database (IWGSC, https://www.wheatgenome.org). Scripts written in R program were used to identify all the functionally annotated candidate genes (iwgsc_refseqv1.0_FunctionalAnnotation_v1__HCgenes_v1.0-repr.TEcleaned.TAB) in both intervals, and their transcript abundances obtained from the MACE data of salt-tolerant (*Altay2000*) and salt-sensitive (*Bobur*) wheat genotypes under saline and non-saline conditions were analyzed.

### Promoter region analyses of three candidate gene targets identified

Because *CREs* regulate the transcription of the neighboring genes, promoter regions of three identified salt stress-responsive candidate genes including *NRAMP*-*2*, *TaABCF3 and CSLF1* were analyzed in the two salt-tolerant (*Altay2000* and *UZ*-*11CWA*-*8)* and two salt-sensitive *(UZ*-*11CWA*-*24* and *Bobur*) wheat genotypes for identification of possible regulatory elements for salt stress tolerance in wheat. Specific oligonucleotides for: *NRAMP*-*2 (5′*-*TCCGGGGCGTTGCTTGAA*-*3*′; *5′*-*TCGGAGATGGAGATGGAGACCT*-*3*′; T_a_ = 60 °C, *TaABCF3* (*5′*-CAACCAACTAGAAGCGAGTG-*3′* and *5′*- GGCGATGTAGGAGACGAT-*3′*; T_a_ = 59.0 °C) and *CSLF1* (*5′*- TGTACATAGCGTCCAGATTTG-*3′* and *5′*-ATCGTCATTTCACCAGCAAC-*3′*; *T*_a_ = 55.5 °C) were designed and used for gene fragment amplification and sequencing. The nucleotide sequences of the amplified candidate gene targets were compared and aligned with each other using MotifViz (Fu et al. 2004; http://biowulf.bu.edu/MotifViz) and PlantPAN 2.0 (Chow et al. 2015; http://PlantPAN2.itps.ncku.edu.tw/). In addition, the amino acid sequences of the analyzed candidate genes at coding regions were compared with their corresponding draft sequences obtained from the Ensembl Web site (Kersey et al. 2016), to check for possible allelic variations that may cause structural and functional variations during salinity stress, using MAFFT version 7 (http://mafft.cbrc.jp/alignment/server/) and Sequence Manipulation Suite (Stothard 2000).

## Results

### Phenotypic analysis of the wheat diversity panel

The effects of salt stress on photosynthetic-related and grain quality traits were analyzed via high-throughput phenotypic methods. Substantial genetic variation was observed in all the HTP_chlF_ traits investigated, as reflected by their corresponding mean, standard deviation (SD) and genotypic coefficient of variation (GCV) values under saline and non-saline conditions (Table 3). The GCVs (GCV = √Genetic variance/Average × 100%) ranged from 0.9 in *F*_v_/*F*_m_ to 13.67% in *PI*_*(ABS)*_ under non-saline conditions and from 1.37 in *F*_v_/*F*_m_ to 16.40% in *PI*_*(ABS)*_ under saline conditions. The skewness and kurtosis values indicated that HTP_chlF_ traits were more or less continuously distributed and thus considered to be quantitatively inherited. REML results showed that variation among genotypes and salt stress treatments were significant (*P* < 0.01) on the HTP_chlF_ traits. Application of salt stress adversely affected the HTP_chlF_ traits by decreasing their values from − 1.43% in *F*_v_ to − 37.04% in *PI*_*(ABS)*_, except for *DIo/RC* (+ 9.71%) and *ETo/RC* (+ 8.98%). Further, HTP_chlF_ traits were heritable with *h*^2^ estimates which ranged from 19% in *F*_v_ to 63% in *ETo/RC*, indicating that the observed genotypic differences can be used for mapping ST QTL.

**Table 3:** Statistics of leaf fluorescence and seed quality traits of the 150 wheat diversity mapping panel under control and saline conditions

Traits	G	T	Non-saline					Saline						
			Mean	SD	GCV	Skewness	Kurtosis	Mean	SD	GCV	Skewness	Kurtosis	E (%)	h^2^
*Leaf chlorophyll fluorescence after 24* *days under stress*														
*F*_*v*_	**	**	− 34,990	1770	5.06	0.24	1.16	− 35,338	2409	6.82	− 0.05	− 0.07	− 1.43	0.19
*F*_*v*_ */F*_o_	**	**	− 2.64	0.08	3.18	− 0.19	0.12	− 2.89	0.11	3.72	− 0.01	− 0.39	− 9.63	0.42
*F*_*m*_ */F*_o_	**	**	− 2.64	0.08	3.18	− 0.19	0.12	− 2.89	0.11	3.72	− 0.01	− 0.39	− 9.63	0.42
*F*_*v*_ */F*_*m*_	**	**	− 0.72	0.01	0.90	− 0.33	0.29	− 0.74	0.01	1.37	− 4.14	33.00	− 2.51	0.33
*ABS/RC*	**	**	3.99	0.08	2.09	0.37	0.50	3.83	0.09	2.47	1.25	6.75	− 3.89	0.11
*DIo/RC*	**	**	1.00	0.05	4.57	− 0.47	4.50	1.10	0.08	8.46	6.68	64.79	+ 9.71	0.10
*ETo/RC*	**	**	1.25	0.08	6.45	0.12	0.27	1.36	0.09	6.58	0.20	− 0.22	+ 8.98	0.63
*TRo/RC*	**	**	2.88	0.05	1.59	− 0.02	0.02	2.84	0.05	1.59	− 0.28	0.17	− 1.60	0.12
*PI*_*(ABS)*_	**	**	− 0.54	0.07	13.67	0.30	0.40	− 0.74	0.12	16.40	0.66	− 0.10	− 37.04	0.30
*Seed grain quality of seed harvested from Karshi field evaluations*														
*GPC*	**	**	12.18	1.10	9.00	0.16	− 0.40	13.77	0.99	7.21	− 0.03	0.35	+ 13.05	0.89
*GSC*	**	**	72.10	1.09	1.52	− 0.42	0.16	71.26	1.11	1.56	− 0.18	0.05	− 1.17	0.93
*NDF*	**	ns	16.84	0.90	5.33	− 0.10	− 0.12	17.01	1.05	6.19	− 0.13	0.11	+ 1.01	0.87
*CFC*	**	**	2.37	0.19	8.03	− 0.25	− 0.27	2.34	0.20	8.44	0.23	0.29	− 1.27	0.98
*Shoot ion concentrations after 24* *days under salt stress (g/100* *g sample dry weight)*														
*Shoot K* ^+^	ns	–	–	–	–	–	–	6.00	1.86	31.05	− 0.04	− 0.72	–	0.10
*Shoot Na* ^+^	**	–	–	–	–	–	–	1.21	0.36	30.24	1.95	8.40	–	0.84
*Shoot K* ^+^ */Na* ^+^	**	–	–	–	–	–	–	5.33	2.02	37.88	0.19	− 0.32	–	0.63

*G* genotypic effect, *T* salt treatment effect, *SD* standard deviation, ***GCV*** genetic coefficient of variation; *E* effect of salt stress on the traits; *ns* nonsignificant effect; – not available; *F*_*v*_ maximal variable fluorescence; *F*_*v*_*/F*_o_ efficiency of the water-splitting complex on the donor side of PSII; *F*_*v*_*/F*_*m*_ maximum quantum yield of PSII within light-adapted; ***ABS/RC*** effective antenna size of an active reaction center (RC); DIo/RC effective dissipation in an active RC; ETo/RC electron transport in an active RC; TRo/RC maximal trapping rate of PSII; PI_(ABS)_ performance index on absorption basis; *GPC* seed protein content; *GSC* seed starch content; *NDF* seed neutral detergent fiber; *CFC* seed crude fiber

**Significant effect at the 0.01 level (2-tailed)

To characterize the effect of salinity on GQ, several grain parameters were defined. All the evaluated GQ traits (except NDF) were strongly influenced by genotype, salt stress and their interaction (Table 3). Substantial variation that could allow for genetic characterization of salt stress responses was observed in GQ traits. The GCVs were: 9.00% and 7.21% for GPC, 8.03% and 8.44% for CFC, 5.33% and 6.19% for NDF, and 1.52% and 1.56% for GSC under non-saline and saline conditions, respectively. The GQ traits followed a continuous distribution in both saline and non-saline-stress field conditions. Field salinity increased GPC by 13.05% and NDF by 1%, but decreased the GSC and CFC by 1.2% and 1.3%, respectively, while *h*^2^ estimates for QG traits were highest in CFC (98%), followed by GSC (93%), GPC (89%) and NDF (87%).

The majority of the STI estimates of HTP_chlF_ traits were significantly correlated (*P* ≤ 0.01) among each other (Table 4**)**, with the strongest correlations (*r*) found between *F*_v_*/F*_o_ and *F*_v_*/F*_m_ (*r* = 0.87**), *ABS/RC* and *DIo/RC* (*r* = 0.83**), and *F*_v_*/F*_o_ and *PI*_*(ABS)*_ (*r* = 0.82**). The correlation values among GQ traits indicated a large but negative correlation between GPC and GSC (*r* = − 0.80**). In addition, the *shoot K*^+^*/Na*^+^ ratio showed moderate (*P* ≤ 0.01) correlation with *ABS/RC*, *TRo/RC*, *F*_v_, *F*_v_*/F*_o_ and *PI*_*(ABS)*_. Similarly, *F*_v_ correlated negatively and positively with GPC (*r* = − 0.24**; *P* ≤ 0.01) and GSC (*r* = 0.26**; *P* ≤ 0.01), respectively.

**Table 4:** Pearson’s correlation based on the genotype mean (150 wheat diversity panel) among the salt tolerance indices (STIs) traits of the measured leaf chlorophyll fluorescence, *shoot K*^+^*/Na*^+^ ratio and seed quality traits

	ABS/RC	DIo/RC	EToRC	TRo/RC	*F*_v_	*F*_v_/*F*_o_	*F*_v_/*F*_m_	PI_(ABS)_	Shoot K^+^/Na^+^	NDF	CFC	GPC	GSC
ABS/RC	1												
DIo/RC	.833**	1											
ETo/RC	− .189*	− .308**	1										
TRo/RC	.751**	.321**	.160*	1									
F_v_	.211**	0.02	.187*	.439**	1								
F_v_/F_o_	− .720**	− .563**	.482**	− .497**	0.115								
*F*_v_/*F*_m_	− .586**	− .534**	.372**	− .301**	0.129	.870**	1						
PI_(ABS)_	0.613**	0.511**	.− 811**	0.379**	0.039	.815**	.590**	1					
Shoot K^+^/Na^+^	.226**	0.144	− 0.097	.286**	.279**	.245**	− 0.152	0.228**	1				
NDF	− 0.128	− 0.146	0.026	− 0.048	0.061	0.153	.206*	0.064	− 0.11	1			
CFC	− 0.053	− 0.082	0.042	− 0.015	− 0.011	0.045	− 0.018	0.049	− 0.068	0.091	1		
GPC	− 0.043	0.003	− 0.001	− 0.112	− .236**	− 0.06	− 0.073	0.03	− 0.119	− .178*	− .391**		
GSC	0.009	− 0.025	0.094	0.095	.255**	0.109	0.129	0.064	0.081	.170*	.234**	− .802**	1

*ABS/RC* effective antenna size of an active reaction center (RC), *DIo/RC* effective dissipation in an active RC, ETo/RC electron transport in an active RC, TRo/RC maximal trapping rate of PSII, *F*_*v*_ maximal variable fluorescence, *F*_*v*_*/F*_o_ efficiency of the water-splitting complex on the donor side of PSII, *F*_v_*/F*_*m*_ maximum quantum yield of PSII within light-adapted, *PI*_*(ABS)*_ performance index on absorption basis, NDF neutral detergent fiber, *CFC* crude fiber, *GPC* protein content, GSC starch content

*,**Correlations are significant at the 0.05 level (2-tailed) and 0.01 level (2-tailed), respectively

### Identification of QTL for ST in the diversity panel

**Fig. 1:**
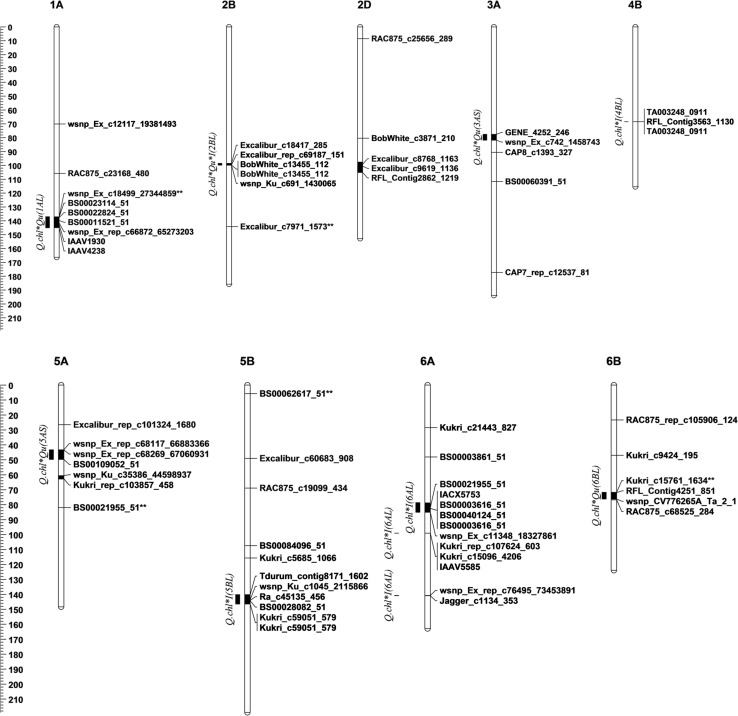
Genetic map of wheat showing the location of the genetic variants (SNP markers) associated with the chlorophyll fluorescence (in green), *shoot K*^+^ and *shoot Na*^+^ (in blue) and seed quality (in red) salt tolerance traits in the 150 wheat genotype diversity panel. Each bar denotes the associated chromosomes, and the black-shaded regions are the associated chromosomal regions (SNP clusters) as defined by the genome LD. The QTL names for each SNP cluster have been presented in the left side the chromosomes

**Fig. 2:**
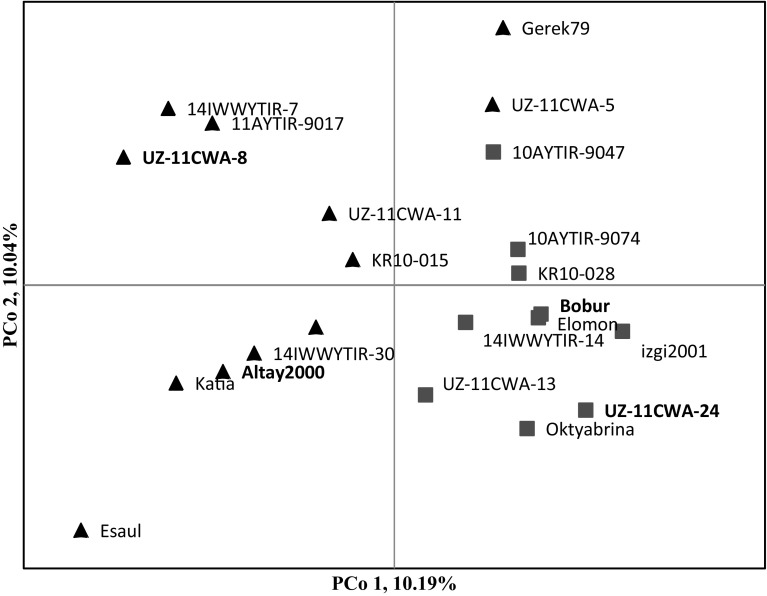
Principal coordinates analysis (PCoA) plot using a genetic distance matrix (GenAlEx 6.5) estimated with data from 115 associated polymorphisms of the salt-tolerant (black color/triangular shaped) and salt-sensitive (red color/squared shape) wheat genotypes previously identified among the studied population. The genotypes (in bold) were used to perform the gene expression and promoter region/cis-element analyses

The goal was to identify chromosomal regions and loci harboring genetic factors associated with salt stress response among the 150 wheat genotype diversity panel. Results identified a total of 106 SNP loci linked with STIs of HTP_chlF_, shoot ionic and GQ ST traits (Fig. 1; Table S1), and they explained between 0.12 and 63.44% of the observed phenotypic variance (PVE). The PCoA performed based on the detected genetic variants accurately separated salt-tolerant and salt-sensitive wheat genotypes into two groups (Fig. 2). Salt-tolerant genotypes were mostly positioned on the left, whereas salt-sensitive lines were distributed on the right side of the plot. The first three PCos accounted for 28.97% of the PVE, and the major genetic contributors to the observed PVE for salt stress response were detected on chromosomes 1AL (at 141.53–144.94 cM), 2BL (at 155.41 cM), 2DL (at 97.42–105.13 cM), 4AS (at 43.39 cM), 4BL (at 68.45 cM), 6AL (at 99.04 cM) and 7AL (at 126.8 and 148.43 cM).

To investigate the pattern and the likely causal relationship among the traits with respect to salt stress response, the effect of all linked SNP loci within each LD-defined genetic interval “*SNP clusters*” was considered to be emanating from single or few genes in high LD. Thus, the 106 associated loci were resolved into 16 unique SNP clusters across the wheat genome, out of which 10 showed strong genetic association with at least two of the independent ST traits evaluated (Table 5; Fig. 1). The SNP cluster on 2BL (PVE = 3.5–8.3%) which spanned an interval of 1.34 cM strongly influenced ST in *F*_v_*/F*_m_, *shoot Na*^+^ uptake, *shoot K*^+^*/Na*^+^ ratio and GPC ST traits. Four SNP clusters, notably on chromosomes 1AS, 3AS, 5AS, and 6BL, were linked with HTP_chlF_ and GQ ST traits, while an additional four SNP clusters on 5BL and 6AL strongly influenced both the HTP_chlF_ and shoot ionic (S_K_^+^–_Na_^+^) ST traits. Previous studies have shown that several SNP clusters detected in this study are in genetic regions known to carry salt stress response QTL (Table 5), while novel QTL regions (“*SNP clusters*”) were also identified.

**Table 5:** Associated SNP clusters and the QTL/genes previously identified within the associated genomic region

QTL	SNP cluster	cM	Associated traits	References
*QTL regions detected for HTP*_*chlF*_, *K*^+^*:Na*^+^*uptake and GQ traits*				
Q.chl*Qu*I(2BL)	98.53–99.87	1.34	*F*_v_*/F*_m_, *Shoot Na*^+^, CFC, *Shoot K*^+^*/Na*^+^	*F*_m_, *F*_v_*/F*_m_, *F*_v_*/Fo* (Zhang et al. 2010b, Li et al. 2012b; *qChlN*-*2B* (Li et al. 2010); *Shoot Na*^+^ (Genc et al. 2010), seed dormancy and PHS loci (Chao et al. 2016), ST_DRW and ST_GY (Oyiga et al. 2018)
*QTL regions detected for HTP*_*chlF*_ *and GQ traits*				
Q.chl*Qu(1AL)	137.12-142.62	5.50	*ABS/RC*, *Dio/RC*, GPC, GSC	*Fo* (Zhang et al. 2010b)
Q.chl*Qu(2DS)	8.52	0	*F*_m_*/Fo*, *F*_v_/*Fo*, *CFC*	*F*_m_ (Zhang et al. 2010b), tenacious glume locus *Tg*-*D1* (Okamoto et al. 2012), grain dormancy (Tan et al. 2006), qGY2Da (Zhang et al. 2009), *Rht8* and *Ppd*-*D1* (Pestsova and Röder 2002; Gasperini et al. 2012)
Q.chl*Qu(3AS)	77.57–81.82	4.25	*TRo/RC*, GPC	HKT gene (Huang et al. 2008)
Q.chl*Qu(5AS)	43.27–49.73	6.46	*ABS/RC*, NDF	*qChlN*-*5A* (Li et al. 2010), *QPext.upm5AS* (Kerfal et al. 2010); LS (Genc et al. 2010); *QSkhard.mna*-*5A.1* (Tsilo et al. 2011); HKT gene (Huang et al. 2008)
Q.chl*Qu(6BL)	71.76–76.20	4.44	*DIo/RC*, NDF, CFC, GPC	*F*_v_*/Fo*, *F*_v_*/F*_m_, *Chl a*, *Chl a *+* b* (Li et al. 2012); grain protein (Prasad et al. 2003); *QFn.sdau*-*6B* (Sun et al. 2008)
*QTL regions detected for HTP*_*chlF*_ *and K* ^*+*^ *:Na* ^*+*^ *uptake*				
Q.chl*Qu(4BL)	68.45	0	*Shoot K*^+^*/Na*^+^, *ABS/RC2*, *TRo/RC*	–
Q.chl*I(5BL)	140.17–146.48	6.31	*F*_v_, *Shoot Na*^+^, *Shoot K*^+^*/Na*^+^	–
Q.chl*I(6AL)	81.96–85.07	3.11	*ABS/RC*, *DIo/RC*, *Shoot Na*^+^	–
Q.chl*I(6AL)	90.04	0	*ABS/RC*, *DIo/RC*, *Shoot Na*^+^	*F*_m_, *F*_v_*/F*_m_ (Li et al. 2012), Shoot Na^+^ (Genc et al. 2010)
Q.chl*I(6AL)	140.7	0	*F*_v_, *Shoot Na*^+^	*Fo* (Li et al. 2012)

PHS preharvest sprouting, *LS* leaf symptoms, *QPext.upm5AS* dough extensibility, *QFn.sdau*-*6B* QTL for falling number (starch trait), *QSkhard.mna*-*5A.1* (Xgwm339–Xbarc311, a QTL for endosperm texture), *ST_DRW and ST_GY* salt tolerance trait indices for dry root weight and grain yield, respectively

### Single SNP loci showing genetic effects on multiple ST traits

The goal was to identify SNP loci exhibiting pleiotropic effects on multiple ST traits. We anticipated that the network of traits sharing genetic association to common SNPs and/or genes may offer some clues as to the underlying shared genetic and molecular mechanisms of salt stress response in wheat. Results from this study showed that a total of 14 SNP loci conferred pleiotropic effects on several ST phenotypes (Table 6). Among them is the locus at 90.04 cM (PVE ≤ 14.7%) on chromosome 6AL which exhibited the most influence with the highest number of ST traits: *ABS/RC*, *DIo/RC*, *shoot Na*^+^ uptake and *shoot K*^+^*/Na*^+^ ratio. Similarly, the SNP locus at 99.80 cM (PVE ≤ 8.3%) on 2BL controlled salt stress response of three ST traits: *F*_v_*/F*_m_, CFC, and *shoot Na*^+^ uptake, while the SNP locus at 82.38 cM (PVE ≤ 14.9%) on chromosome 6AL had a strong effect on the ST traits for specific energy fluxes (per reaction center) for energy absorption (*ABS/RC)* and dissipation (*DIo/RC)*, and *shoot Na*^+^ uptake. In addition, the locus at 118 cM on 7AL affected *DIo/RC*, *F*_v_*/F*_m_, and *shoot K*^+^*/Na*^+^ ratio, whereas the SNP locus at 71.76 cM (PVE ≤ 4.1%) on chromosome 6BL influenced only GQ traits: NDF, CFC, and GPC. A locus at 68.45 cM on chromosome arm 4BL associated with three ST traits including specific energy fluxes (per reaction center) for energy absorption (*ABS*/*RC*) and trapping (*TRo/RC*) and *shoot K*^+^*/Na*^+^ ratio. Further analysis indicated that most of the SNP markers with pleiotropic effects on the ST traits were located in the coding regions of known photosynthetic and abiotic/salt stress-related candidate genes (Table 6). The presence of SNPs and sequence variations in the gene coding region can influence gene structure and function resulting in altered plant responses to stress.

**Table 6:** SNP loci showing multiple effects on the independent ST traits and the corresponding underlying genes and their biological functions as reported in Berardini et al. (2015)

Chr.	cM	*R* ^2^	Associated ST traits	Associated candidate genes	Biological functions
1AL	137.12	≤ 4.6	GPC, GSC	*DnaJ homolog subfamily C member* 2 [*Triticum urartu*]	Protein homeostasis and protein complex stabilization, chloroplast development and stabilization of PSII complexes, amyloplast organization, cell division, embryo development ending in seed dormancy, embryonic axis specification, endocytosis, endosome organization, late endosome to vacuole transport, negative gravitropism, phototropism, protein folding, protein targeting to vacuole, response to starvation, vacuole organization
2DS	8.52	≤ 3.5	*F*_m_*/F*_o_, *CFC*	*Bidirectional sugar transporter SWEET6b* [*Triticum urartu*]	Carbohydrate transport, protein homooligomerization
2BL	99.80	≤ 8.3	*F*_v_*/F*_m_, CFC, *Shoot Na*^+^	*Putative mixed*-*linked glucan synthase* 3 [*Aegilops tauschii*]	Cellulose biosynthetic process, cell wall organization
2BL	144.16	≤ 18.3	*Shoot Na*^+^, *Shoot K*^+^*/Na*^+^	*Dihydroorotate dehydrogenase (quinone)*, *mitochondrial* [*Triticum urartu*]	Dihydroorotate dehydrogenase catalyzes the fourth step of pyrimidine biosynthesis
4AS	43.39	≤ 9.24	*F*_v_, *Shoot K*^+^*/Na*^+^	*Putative methionyl*-*tRNA synthetase* [*Triticum urartu*]	Methionine-tRNA ligase activity, tRNA binding, aminoacyl-tRNA ligase activity, nucleotide binding, ATP binding, response to cadmium ion
4BL	68.45		*ABS*/*RC*, *TRo/R*, *Shoot K*^+^*/Na*^+^ ratio	*NADH dehydrogenase complex (plastoquinone) assembly (Metal transporter*- *NRAMP*-*2*)	Photosynthetic electron transport in photosystem I, metal ion transporter/homeostasis and inorganic anion transport (Kriventseva et al. 2014)
5AL	81.96	≤ 7.3	*DIo/RC*, *F*_v_*/F*_m_	*Cysteinyl*-*tRNA synthetase* [*Triticum urartu*]	Cysteine-tRNA ligase activity, nucleotide binding, ATP binding, response to cadmium ion, cysteinyl-tRNA aminoacylation
5BL	146.48	≤ 13.3	*Shoot Na*^+^, *Shoot K*^+^*/Na*^+^	*Defective In Exine Formation* 1 (LOC100842013), mRNA	Cell wall organization, pollen exine formation, pollen wall assembly
5BS	5.70	≤ 4.8	*F*_v_, *ETo/RC*	*High affinity cationic amino acid transporter* 1 [*Triticum urartu*]	l-Arginine import, L-glutamate import, amino acid transmembrane transport, basic amino acid transport
6AL	82.38	≤ 14.9	*ABS/RC*, *DIo/RC*, *shoot Na*^+^	Universal stress protein A-like protein [*Triticum urartu*]	Response to cold, response to stress, functions as a molecular chaperone under heat shock and oxidative stress conditions
6AL	90.04	≤ 14.7	*ABS/RC*, *DIo/RC*, *shoot Na*^+^, *shoot K*^+^*/Na*^+^	*Myosin*-*J heavy chain* [*Aegilops tauschii*]	Motor activity, ATP binding, actin filament-based movement
6BL	71.76	≤ 4.1	NDF, CFC, GPC	*Galactoside 2*-*alpha*-*L*-*fucosyltransferase* [*Aegilops tauschii*]	Cell wall biogenesis, cell wall organization, plant-type cell wall biogenesis, xyloglucan biosynthetic process
7AL	118.4	≤	*DIo/RC*, *F*_v_*/F*_m_, *Shoot K*^+^*/Na*^+^	*LRR receptor*-*like serine/threonine*-*protein kinase*	Embryo development, endodermal cell differentiation, establishment of protein localization, plant epidermis development, potassium ion homeostasis, protein phosphorylation, regulation of cell division, regulation of cell fate specification, regulation of endodermal cell differentiation, regulation of root development, regulation of root morphogenesis, response to wounding, specification of plant organ axis polarity, transmembrane receptor protein tyrosine kinase signaling pathway, plant-type hypersensitive response
7BL	155.41	≤ 3.6	*F*_v_*/F*_m_, *F*_v_*/F*_o_	*Putative serine/threonine*-*protein kinase Cx32*, *chloroplastic* [*Triticum urartu*]	ATP binding, photosystem II stabilization, protein phosphorylation

### Annotation and ontological classifications of the associated SNP loci

Most of the putative candidate genes identified belong to categories of genes involved in abiotic stress and salt stress response. They include those involved in stress response (27.9%), ion/metal transport (12.8%), carbohydrate and energy metabolisms (10.5%) and photosynthetic (10.5%) activities. Others include genes in high sequence homology with the SNP markers identified that function in the protein repair maintenance (10.5%) and transcription factor (10.5%) pathways. Few of the SNP marker sequences identified coded for genes involved in reactive oxygen species (ROS) scavenging (4%) and protein translation (2%) activities. The ontological analyses of the mapped genetic variants provided evidence that the SNP loci identified are coding for salt stress response (data not shown).

The SNP marker at 90.04 cM that exhibited pleiotropic effects on *ABS/RC*, *DIo/RC*, *shoot Na*^+^ uptake and *shoot K*^+^*/Na*^+^ ratio on 6AL is homologous to *OPAQUE1* that encodes a *Myosin XI motor protein* (myo), while the locus at 99.80 cM on 2BL showed high sequence homology to putative *mixed*-*linked glucan synthase 3.* The SNP marker at 82.38 cM on 6AL associated with *ABS/RC*, *DIo/RC* and *shoots Na*^+^ uptake showed a high sequence identity with *universal stress protein* A-*like protein* (USP). The SNP locus at 71.76 cM on 6BL closely linked to NDF, CFC and GPC and was located within the domain of *Galactoside 2*-*alpha*-*L*-*fucosyltransferase*, while the *LRR receptor*-*like serine/threonine*-*protein kinase* transcript was identified at 118 cM (which is strongly influenced *DIo/RC*, *F*_v_*/F*_m_, *shoot K*^+^*/Na*^+^ ratio) on 7AL. Additionally, the SNP marker on 6AL at 140.87 cM associated with *F*_v_ and *shoot Na*^+^ uptake showed high sequence identity with *T*a*ABCF3* transporter. The SNP markers at 68.45 cM on 4BL associated with *shoot K*^+^*/Na*^+^ ratio, *ABS*/*RC*, and *TRo/RC* revealed high sequence identity with *NADH dehydrogenase complex* (plastoquinone) assembly (*NRAMP*-*2*) involved in the photosynthetic electron transport in photosystem I and metal ion transporter/homeostasis. The biological functions of these candidate genes are presented in Table 6.

### Expression analyses of genes associated with the detected ST SNP marker

**Fig. 3:**
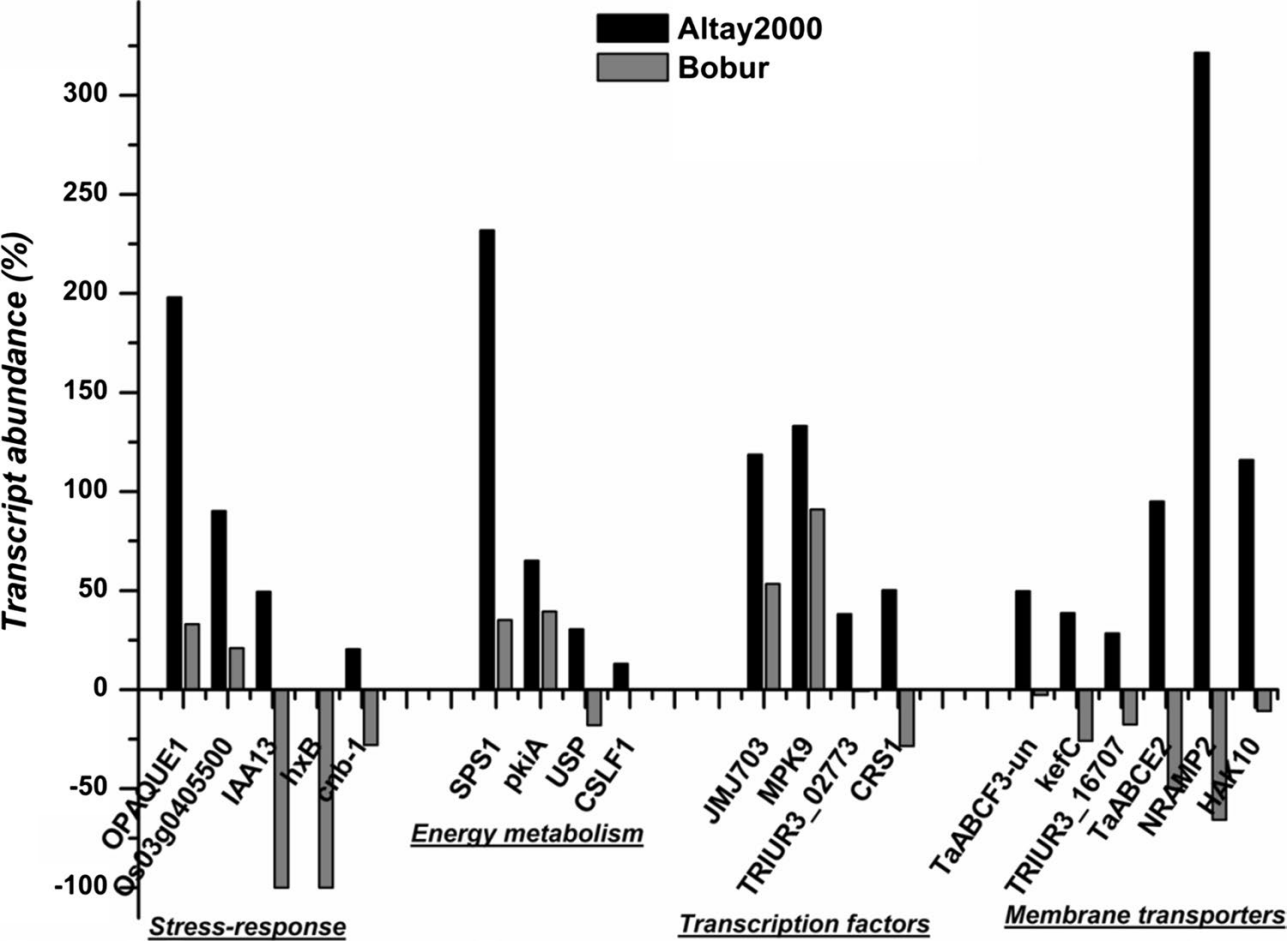
Effect of salt stress on some of the identified gene transcript abundance (% change to control) between salt-tolerant genotype (*Atlay2000*, in black) versus salt-sensitive genotype (*Bobur*, in gray) after 24 days of stress. *OPAQUE1 *= Myosin-J heavy chain; *Os03g0405500 *= Probable nucleoredoxin 1-1; *IAA13 *= Auxin-responsive protein; *hxB *= Molybdenum cofactor sulfurase; *cnb*-*1 *= Calcineurin subunit B; *SPS1 *= Probable sucrose-phosphate synthase 1; *pkiA *= Pyruvate kinase; *USP *= UDP-sugar pyrophosphorylase; *CSLF1* = Putative mixed-linked glucan synthase 1; *JMJ703 *= Lysine-specific demethylase; *MPK9 *= Mitogen-activated protein kinase 9; *TRIUR3_02773* = Putative serine/threonine-protein kinase *Cx32*, chloroplastic; *CRS1 *= Chloroplastic group IIA intron splicing facilitator; *TaABCF3-un* = ABC transporter F family member 3 protein; *kefC *= Glutathione-regulated potassium-efflux system protein; *TRIUR3_16707 *= Multiple C2 and transmembrane domain-containing protein 1; *TaABCE2* = ABC transporter E family member 2, *NRAMP2 *= NADH dehydrogenase complex (plastoquinone) assembly (Metal transporter- *Nramp2*); *HAK10 *= Potassium transporter 10

To gain understanding whether the underlying candidate genes are responsive to salt stress, we investigated the expression levels and the dynamics of 28 associated gene transcripts in the third leaves of *Altay2000* (salt-tolerant) and *Bobur* (salt-sensitive) wheat genotypes for up to 24 days under saline (100 mM NaCl) and non-saline conditions. The transcript amounts were obtained from genome-wide gene expression profiling performed using the MACE transcriptome approach. As shown in Table 7 and Fig. 3, the analyzed candidate genes showed differential expression between the salt-tolerant and salt-sensitive wheat genotypes. Except in few instances, all the genes were up-regulated and/or highly expressed (relative to the non-saline condition) in *Altay2000* and down-regulated in *Bobur*. For instance, *OPAQUE1* was up-regulated by +198.0% in *Altay2000* and + 32.9% in *Bobur*, while *NRAMP*-*2* was also up-regulated by + 321.5% in *Altay2000* and down-regulated by − 65.8% in *Bobur*. The activity of the *T*a*ABCF3* transporter increased by + 47.7% and + 2.9% in the salt-tolerant and salt-sensitive wheat genotypes, respectively, and *sucrose-phosphate synthase* increased by + 231.9% in *Altay2000* and + 35.2% in *Bobur*.

**Table 7:** Relative transcript abundance of 28 candidate genes harboring the associated SNP markers

Associated traits	Gene annotation of the associated SNP loci	UniProt ID	Transcript abundance (%)		RE
			Atlay2000	Bobur	
*F*_v_/F_o_	Callose synthase 2 OS = *Triticum urartu*	M7YGW	0	− 58.35	+,−
Crude fiber	Lysine-specific demethylase JMJ703 OS = *Oryza sativa* subsp. *japonica*	Q53WJ1	118.71	53.33	+,+
Protein	Mitogen-activated protein kinase 9 OS = *Oryza sativa* subsp. *japonica*	Q6L5D4	133.11	90.94	+,+
ABS/RC	Structural maintenance of chromosomes protein 3 OS = *Schizosaccharomyces pombe*	O42649	45.74	− 8.30	+,−
Crude fiber	Metallothionein-like protein 1 OS = *Triticum aestivum*	P43400	13.00	8.90	+,+
Shoot Na^+^	Dihydroorotate dehydrogenase (quinone) OS = *Azorhizobium caulinodans*	A8HZX8	− 43.09	− 4.77	−,−
Shoot Na^+^, Rohfaser	Putative alanine aminotransferase OS = *Schizosaccharomyces pombe*	Q10334	− 7.76	58.78	−,+
Crude fiber	Glutathione-regulated potassium-efflux system protein KefC OS = *Enterobacter* sp.	A4W6F3	38.61	− 25.89	+,−
ABS/RC	Probable nucleoredoxin 1-1 OS = *Oryza sativa* subsp. *japonica*	Q7Y0E8	90.16	20.87	+,+
Moisture content	Multiple C2 and transmembrane domain-containing protein 1 OS = *Triticum urartu*	M7YGD3	28.42	− 17.64	+,–
ETo/RC	Probable sucrose-phosphate synthase 1 OS = *Oryza sativa* subsp. *indica*	A2WYE9	231.83	35.15	+,+
Shoot K^+^/Na^+^, ABS/RC2, TRo/RC	NADH dehydrogenase complex (plastoquinone) assembly (Metal transporter-NRAMP2)	Q10Q65	321.52	− 65.78	+,−
Moisture content	Auxin-responsive protein IAA13 OS = *Oryza sativa* subsp. *indica*	A2XLV9	49.43	− 100.00	+,−
ABS/RC	NAD(P)H-quinone oxidoreductase subunit L OS = *Nostoc* sp.	Q8YMW5	− 25.21	19.65	−,+
Seed hardness	Molybdenum cofactor sulfurase OS = *Neosartorya fischeri*	A1CX75	0	− 100.00	+,−
Shoot Na^+^	Leukotriene A-4 hydrolase homolog OS = *Neurospora crassa*	Q7S785	62.17	40.43	+,+
Crude fiber	Phospholipase D alpha 1 OS = *Zea mays*	Q43270	65.10	316.94	+,+
Shoot Na^+^	UDP-glucose 6-dehydrogenase 2 OS = *Oryza sativa* subsp. *japonica*	B7F958	85.74	− 6.07	+,−
ETo/RC	Pyruvate kinase OS = *Emericella nidulans*	P22360	65.09	39.44	+,+
Shoot Na^+^, Shoot K^+^/Na^+^, Dio/RC, ABC/RC	Myosin-J heavy chain [*Aegilops tauschii*] (OPAQUE1)	O94477	198.03	32.94	+,+
NDF	Potassium transporter 10 OS = *Oryza sativa* subsp. *japonica*	Q67VS5	115.92	− 10.822	+,−
*F*_v_	UDP-sugar pyrophosphorylase OS = *Oryza sativa* subsp. *indica*	A2YGP6	30.51	− 17.96	+,−
*F*_m_/F_o_	Putative serine/threonine-protein kinase Cx32, chloroplastic OS = *Triticum urartu*	M7ZVA6	38.05	− 0.69	+,−
*F*_v_/F_o_	Chloroplastic group IIA intron splicing facilitator CRS1, chloroplastic OS = *Zea mays*	Q9FYT6	50.14	− 28.49	+,−
*F*_m_/F_o_	Sucrose synthase 2 OS = *Oryza sativa* subsp. *japonica*	P30298	− 50.00	819.81	−,+
Crude protein	Mitogen-activated protein kinase 9	Q6L5D4	133.11	90.94	+,−
*F*_v_, Shoot Na^+^	ABC transporter F family member 3	O59672	47.66	2.85	+,+
*F*_v_/*F*_m_, Shoot Na^+^, CFC, Shoot K^+^/Na^+^	Putative mixed-linked glucan synthase 1	Q6ZF89	13	0	+,−

RE, effect of salt on the gene expression in relation to non-saline condition; +,− = gene transcript abundance was up-regulated in *Atlay2000* but down-regulated in *Bobur*; −,+ = gene transcript abundance was down-regulated in *Atlay2000* but up-regulated in *Bobur*; ++ = gene transcript abundance was up-regulated in both *Atlay2000* and *Bobur*; − = gene transcript abundance was up-regulated in both *Atlay2000* and *Bobur*

The salt stress response kinetics of *Altay2000* and *Bobur* at 2 h, 11 days and 24 days under saline stress and non-saline conditions were analyzed using sigmoidal nonlinear statistics (Figure S1). Results indicated that the temporal patterns of the expressed transcripts change over time during salt stress exposure and varied between the salt-tolerant and salt-sensitive wheat genotypes. *Altay2000* and *Bobur* exhibited differential expression signatures at 2-h stress time period, with the latter showing mostly higher transcript amounts. The observed trends were maintained for few (0–5 days) days and thereafter changed at 11 days of salt stress with the transcript amount induced by salinity in *Altay2000* rising steadily and exponentially and mostly decreased in *Bobur*. Further, genes such as *OPAGUE1*, *molybdenum cofactor sulfurase* (*hxB*) and *NRAMP*-*2* exhibited an early (0–5 days) salt stress response, while *mitogen*-*activated protein kinase 9* (*MPK9*) was differentially expressed after 15 days of salt stress application.

**Fig. 4:**
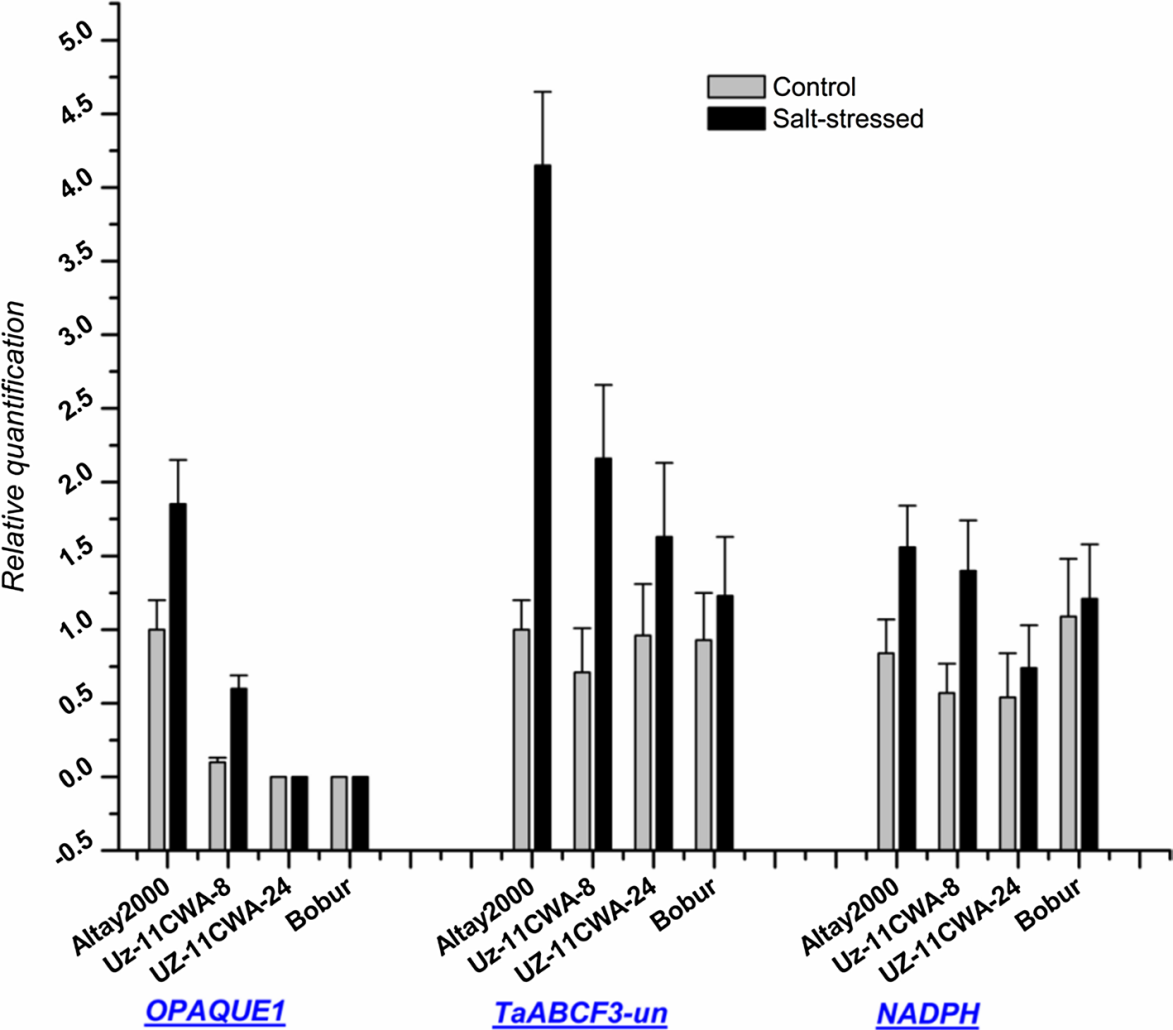
The qRT-PCR analyses of ***OPAQUE1*** (on 6AL): *myosin-J chain*, ***TaABCF3*-**_***un***_ (on 6AL): ABC transporter F family member 3 and ***NADPH*** (on 5AS): NAD(P)H-quinone oxidoreductase subunit L, chloroplastic in leaves of two salt-tolerant (*Atlay2000* and *UZ*-*11CWA*-*8*) and salt-sensitive (*UZ*-*11CWA*-*24* and *Bobur*) after 30 days in non-saline (gray) and saline (black) conditions. *Efa1.1* and *Efa1.2* genes were used as internal control genes. Bars are the means ± standard error (*n* = 3)

The RT-qPCR performed to analyze the gene expression patterns after an extended period of 30 days under salt stress also revealed increased activities of *OPAQUE1*, *TaABCF3*-_*un*_ and *NAD(P)H*-*quinone oxidoreductase subunit L*, *chloroplastic* in the two salt-tolerant (*Altay2000* and *UZ*-*11CWA*-*8*) genotypes, compared with the two salt-sensitive *(UZ*-*11CWA*-*24* and *Bobur*) genotypes (Fig. 4). The candidate gene *OPAQUE1* was highly expressed in the salt-tolerant genotypes, with no corresponding and meaningful transcriptional changes in the salt-sensitive genotypes. However, salt stress induced greater transcriptional changes in *TaABCF3*-_*un*_ and *NADPH* in both the two salt-tolerant and two salt-sensitive wheat genotypes, although the fold-changes were much higher in the two salt-tolerant genotypes.

### Identification and expression analyses of the genes in the interval surrounding the detected SNP markers

**Fig. 5:**
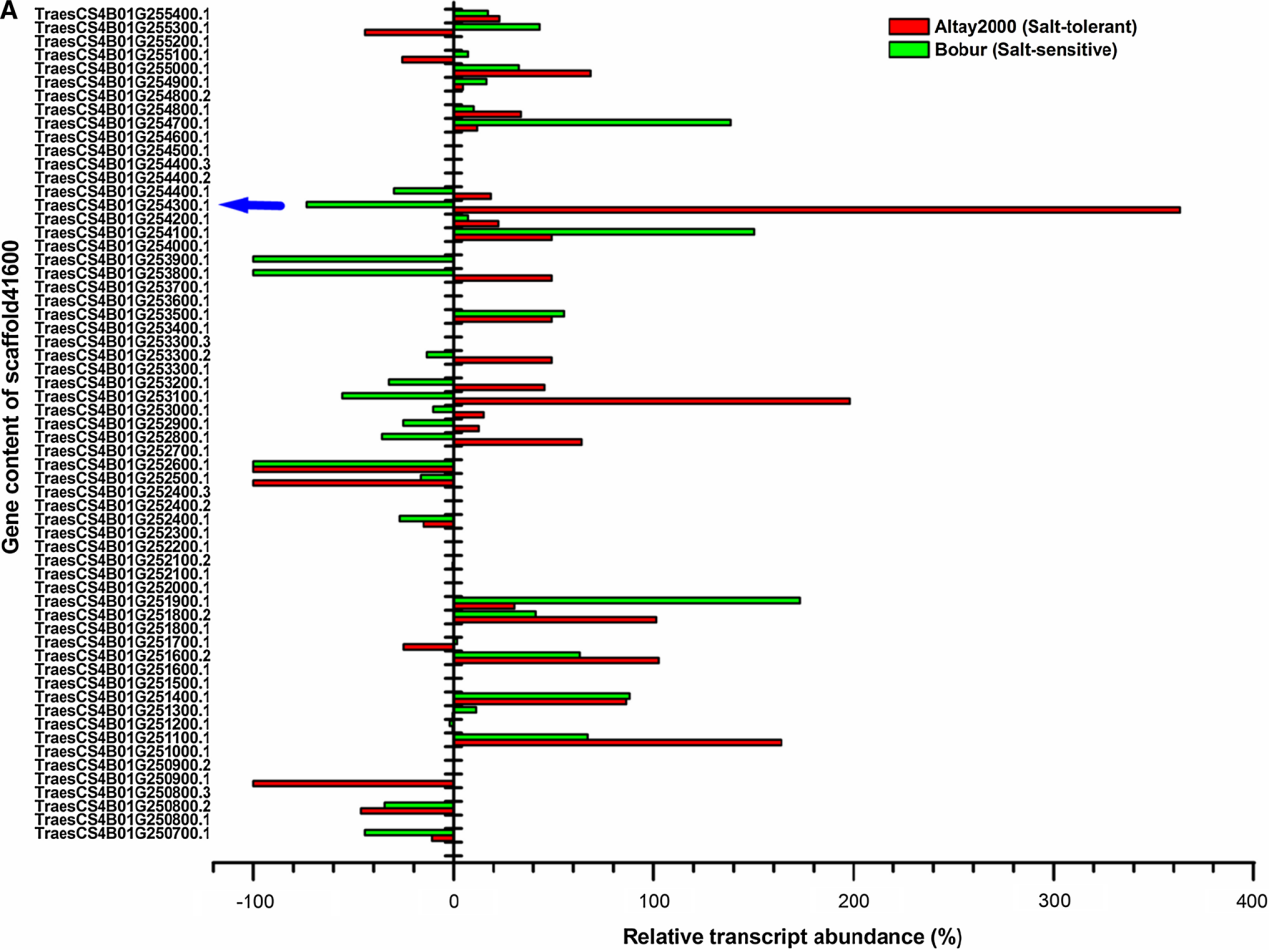
Relative expression levels of all the annotated genes (in their natural order) belonging to s*caffold41600* (~ 13 Mb) due to salt stress in *Altay2000* (salt-tolerant) and *Bobur* (salt-sensitive) genotypes after 24 days. The relative abundance of the genes was determined as a change in transcript amounts after 24 days of salt stress relative to an untreated control. The “thick blue arrow” represents the gene domain carrying the GWAS mapped pleiotropic SNP marker sequences

**Fig. 6:**
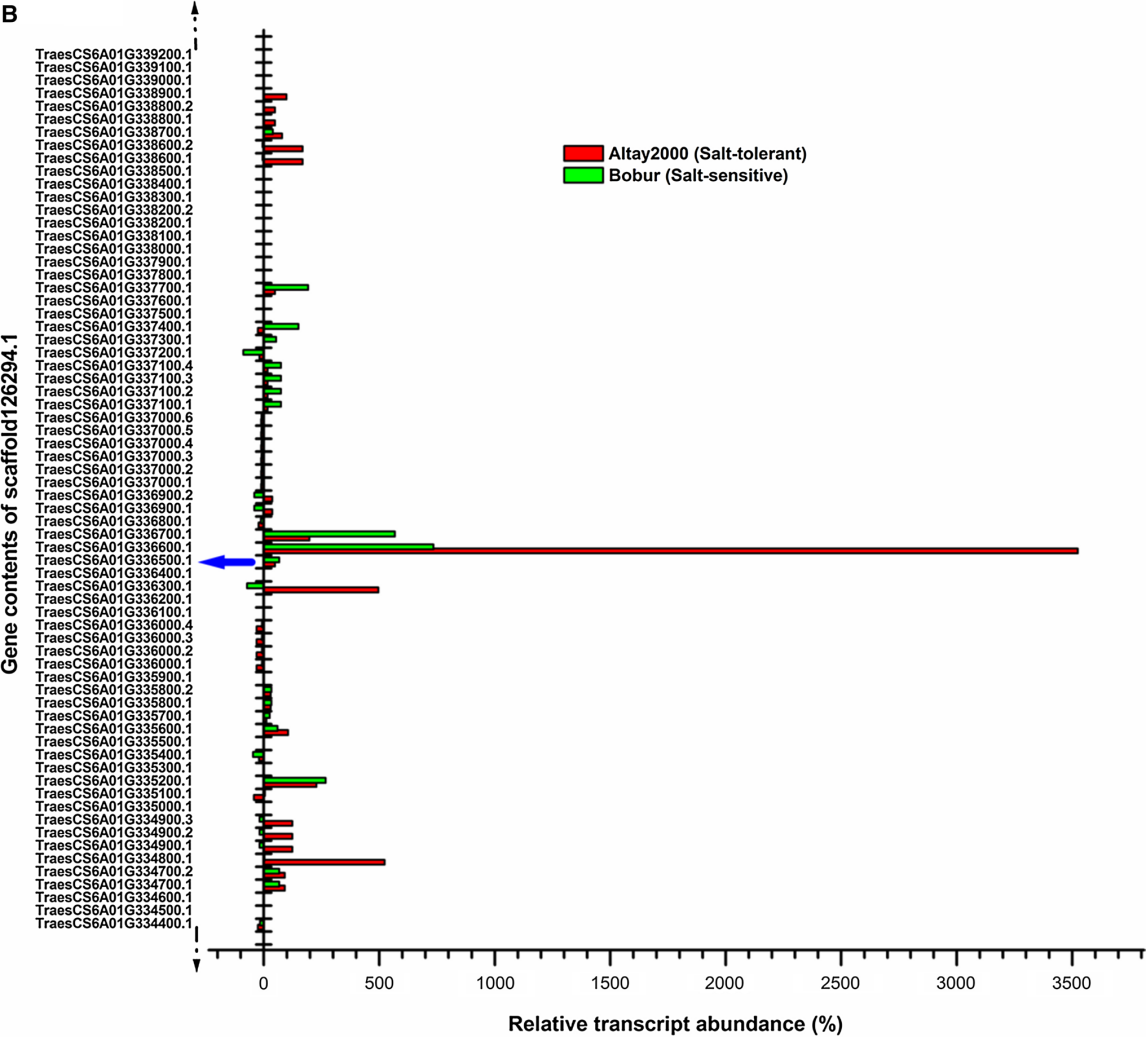
Relative expression levels of all the annotated genes (in their natural order) belonging to *scaffold126294.1* (~ 12 Mb) due to salt stress in *Altay2000* (salt-tolerant) and *Bobur* (salt-sensitive) genotypes after 24 days. The relative abundance of the genes was determined as a change in transcript amounts after 24 days of salt stress relative to an untreated control. The “thick blue arrow” represents the gene domain carrying the GWAS mapped pleiotropic SNP marker sequences

The Blast search in the IWGSC database showed that the genetic interval in LD block within the *scaffold41600* (~ 13 Mb containing 61 ORFs or putative genes) on 4BL contains the SNP marker located at 68.45 cM (Table S2; Fig. 5), whereas the interval covering ~ 12 Mb in *scaffold126294.1* contains 141 candidate genes and corresponds to the chromosomal region containing the pleiotropic SNP marker (at 90.04 cM) on 6AL (Table S3; Fig. 6). Results from this study indicated that *TraesCS4B01G254300.1* which codes for *NRAMP*-*2* was the most highly differentially expressed gene between the salt-tolerant and salt-sensitive genotypes after 24 days when the expressions of all the genes in the associated interval in *scaffold41600* were analyzed. Salinity stress increased the expression of *TraesCS4B01G254300.1* by + 363.4% in *Altay2000* but decreased it by − 73.49% in *Bobur*. Highest transcriptional change differentials were also observed between the salt-tolerant (+ 3524.72%) and salt-sensitive (+ 734.90%) in *TraesCS6A01G334400.1* (tyrosine kinase proteins) which is 317 bp away from *TraesCS6A01G336500.1* (coding for *OPAQUE1*) that contains the SNP locus with pleiotropic effects on ST traits detected on chromosome 6AL.

### Promoter response elements of the underlying salt-tolerant genes

To shed light into the mechanisms of the underlying gene expression regulations, the promoter regions of *NRAMP*-*2*, *OPAQUE1*, *CSLF1* and *TaABCF3* candidate genes were examined for possible detection of potential cis-elements involved in salt stress response using the salt-tolerant and salt-sensitive genotypes (Figures S2–4). The two tolerant genotypes showed unique DNA-binding domains of *TF_motif_seq_0239*/*Dof* (5′-AA[AG]G-3′) and *PBF* (5′-AAAGC-3′) at 99 bp upstream of the *NRAMP*-*2* start codon (Figure S2). However, these motif features were found to be lacking in the two sensitive genotypes but instead were replaced by the *broad*-*complex 3* (5′-TCAACAAAAAC-3′)—and/or *hunchback* (5′-CAACAAAAAC-3′)—DNA-binding domains. The promoter sequence of 950-bp upstream of the *OPAQUE1* gene start codon revealed no differences in the transcription factor binding sites of the contrasting ST wheat genotypes (Data not shown). The analysis of *CSLF1* promoter regions showed genotype-specific motif variation among the wheat genotypes including the *homeodomain motif family*—*bZIP*; *HD*-*ZIP* (5′-GCAATCATAG-3) and *GATA*-*binding* proteins (5′-GATA-3) identified at 581 and 554 bp, respectively, upstream of the gene start codon of *UZ*-*11CWA*-*08*; the *SPI*-*B* (5′-TTCCGCT-3′) and *NF*-*Y* (5′-ATGAACCAATGCATGC-3′) found at positions 618 bp and 540 bp of *UZ*-*11CWA*-*24*, respectively (Figure S3). Motif differences were not detected among the genotypes in *TaABCF3*; however, the translation initiator codon of the gene was 21-bp downstream in *UZ*-*11CWA*-*24* when compared to the other genotypes (Figure S4).

### Structure analysis of the gene coding regions

**Fig. 7:**
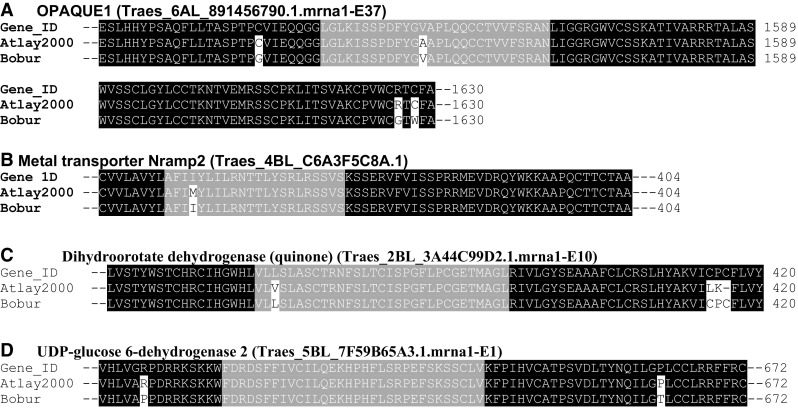
Comparison of the deduced EST amino acid sequence in *Atlay2000* (salt-tolerant) and *Bobur* (salt-sensitive) genotypes with their corresponding draft sequence obtained from Ensembl Genomes database (http://www.ensemblgenomes.org). Black and white colors indicate the identical and mutation sites, respectively, while gray colors represent the regions anchoring the core sequences of the SNP marker identified in this study

Part of the objective of this study was to identify allele sequence differences between salt-tolerant and salt-sensitive genotypes within the candidate genes encompassing the detected pleiotropic SNP loci since their residue sequence pattern and constitution may affect the protein turnover as well as alter the underlying gene structure and function. Comparative sequence analysis revealed several point polymorphisms; some of them were non-synonymous when *Altay2000* and *Bobur* were compared (Fig. 7). Four SNP sites were detected in exon 37 of *OPAQUE1* (Fig. 7a), leading to the following putative changes: Cysteine to Glycine at position 1529 (C1529G), Alanine to Valine at position 1549 (A1549V), Arginine to Glycine at position 1626 (R1626G) and Cysteine to Tryptophan at position 1628 (C1628W). Several polymorphic sites which may have altered the structure and functions of *NRAMP*-*2* (Fig. 7b), *Dihydroorotate dehydrogenase (quinone)* (Fig. 7c**)** and *UDP*-*glucose 6*-*dehydrogenase* 2 (Fig. 7d) were also detected with the mapped SNP loci.

## Discussion

Developing salt-tolerant cultivars is the preferred breeding strategy for improving wheat adaptation to salinity stress. This strategy requires the comprehensive understanding and identification of genetic and molecular mechanisms involved in salt stress response in wheat. Previous studies indicated that the studied wheat diversity panel responded differently to salt stress when yield-related traits across three growth stages and the *shoot Na*^+^ content and *shoot K*^+^*/Na*^+^ ratio of the third leaves were evaluated (Oyiga et al. 2016, 2018). The main goal of this study was to explore genetic variation in the diversity panel to identify genomic regions and candidate genes involved in salt stress response by analyzing simultaneously different plant physiological and grain quality parameters under different salt stress regimes. To the best of our knowledge, this is the first study that simultaneously evaluated the genetic mechanisms of salt stress response in wheat using three independent salt stress parameters. The identification of genetic variants that have pleiotropic effects on the ST traits would support further molecular breeding research efforts toward improving wheat adaptation and tolerance to salinity stress.

### Phenotypic variability among the 150 diversity panel

The studied panel showed substantial (*P* < 0.001) phenotypic variation for salt stress tolerance in all the HTP_chlF_, GQ and shoot ionic traits evaluated (Table 2) with moderate-to-high h^2^ estimates, suggesting that the traits evaluated can be exploited to uncover the genetic architecture governing salt stress response in wheat.

Application of salt stress impacted negatively on the HTP_chlF_ traits including the maximum quantum yield of PSII chemistry (*F*_v_*/F*_m_*)* and specific energy fluxes (per reaction centre) for both absorption (*ABS*/*RC*) and trapping (*TRo/RC*), indicating that the photosynthetic activities of the evaluated germplasm were suppressed under salinity stress. It has been shown that salt stress reduces photosynthetic activities in plants via the inhibition of photosystem II complex (PSII) at both acceptor [QA] and donor sides (oxygen-evolving complex OEC), and destruction of chlorophyll pigments by the accumulation of toxic ions (Chen and Murata 2011; Shu et al. 2012). The reduction observed in *F*_v_*/F*_m_ may be associated with damage to the PSII reaction center (Kadir et al. 2007) and the decrease in the quantum efficiency of PSII photochemistry (Mathur and Jajoo 2014). The dissipation energy (*DIo*/*RC*) and electron flux (*ETo/RC*) per active reaction center increased during salt stress, due to the increased proportion of the inactive reaction centers in the leaves exposed to salt stress (Mehta et al. 2011), resulting in the ineffective exploitation of light energy received in the inactivated reaction centers (Satoh et al. 1983). Demetriou et al. (2007) and Mathur et al. (2013) have shown that the increase in *ETo/RC* in plant leaves during stress is related to the increasing number of inactive centers and the inefficient transfer of electrons from Q_A_ to Q_B._ The performance index of PSII-*PI*_*(ABS)*_, which is the combination of the indexes of *ABS/RC*, *phi_Po* and *psi_0* (Appenroth et al. 2001), decreased under salt stress supporting the earlier results that PSII activities are affected by salt stress.

The analyses of GQ traits have shown that GPC and GSC are increased and decreased, respectively, under field salinity stress. Reports showing the impact of major abiotic stresses on GQ traits abound, but none have reported on effect with salinity stress in wheat. For instance, ozone (O_3_) (Piikki et al. 2008; Zheng et al. 2013) and heat (Farooq et al. 2011) stresses have been shown to increase the GPC in wheat, while the study conducted by Fuhrer et al. (1990) revealed that O_3_ stress decreases the GSC. In rice, the GSC decreased under salinity stress but the GPC increased (Baxter et al. 2011; Thitisaksakul et al. 2015), which is consistent with the findings from this study, implying that salinity stress increases the ability of plants to mobilize N in developing seeds at the expense of the carbon sink and source, resulting in less dry matter accumulation. The decrease in GSC might be connected with the lower plant growth rate—a direct consequence of the photosynthetic limitation in plants, which may have indirectly contributed to a reduction in the sink capacity and less starch deposition in the grains.

### Correlations among ST traits

Correlated traits are of interest in plant breeding because they help to: (i) connect the genetic causes of the pleiotropic action of genes, (ii) understand how selection for one character will produce a simultaneous change in another character, and (iii) determine the relationship between traits and fitness (Sandhu et al. 2013). Our results indicated that the Pearson’s correlation coefficients (*r*) among the salt tolerance indices of HTP_chlF_ trait were in many cases statistically significant, indicating that the salt stress responses via the pathways leading to photosynthesis follow similar pattern. Positive correlations were observed between *shoot K*^+^*/Na*^+^ ratio and some HTP_chlF_ trait indices including *F*_v_, *ABS/RC*, *TRo/RC* and *F*_v_*/F*_o_. High uptake of K^+^ improves plant stomatal movement, energy transfer and photosynthetic activities (Marschner and Marschner’s 2012), while the high *shoot K*^+^*/Na*^+^ ratio is essential for ion homeostasis in salt-stressed plants (Maathuis and Amtmann 1999; Cuin and Shabala 2006; Volkov and Amtmann 2006). Using these correlated HTP_chlF_ traits as a “physiological marker” for indirect measurement of *shoot K*^+^*/Na*^+^ ratio (ion homeostasis) may offer an indirect and rapid approach for evaluating and mapping QTL linked to salt stress responses, as proposed by Li et al. (2014b). The negative correlation (*r* = − 0.82; *P* < 0.01) observed between GPC and GSC in this study has also been reported in wheat by Hucl and Chibbar (1996) and Burešová et al. (2010). Our findings suggest that the correlated HTP_chlF_ traits have a great potential for application in wheat-breeding programs focused on accelerating the QTL mapping and incorporation of ST alleles into cultivated varieties. Together these shorten the generation interval and increase accuracy of selection to reduce the cost of breeding.

### Genome-wide association studies (GWAS)

The genome-wide scan identified 106 SNP markers (PVE = 0.12–63.44%), belonging to 16 LD-defined SNP clusters, associated with ST in the diversity wheat panel. Among them, 60, 23 and 23 SNP markers were associated with HTP_chlF_, shoot ionic (*shoot K*^+^ uptake, *shoot Na*^+^ uptake and *shoot K*^+^*/Na*^+^ ratio) and GQ parameters, respectively. Comparing the SNP locations identified in this study with previous genetic studies, we found that most were mapped proximal to reported QTL and/or genes involved in the salt stress-related responses. The coincidence of the mapped positions supports the hypothesis that the QTL underlying the ST PVE is mostly conserved due to LD and may be under the influence of orthologous genes (Table 5). Interestingly, the PCoA based on the detected SNP markers successfully discriminated the salt-tolerant and salt-sensitive wheat genotypes. The former and latter were mostly distributed in the left and right of the PCoA plot, respectively—an indication that the SNP loci detected in this study are underlying salt stress-responsive candidate genes. One of the promising QTL detected on 2BL chromosome had pleiotropic effects on *F*_v_*/F*_m_, *shoot Na*^+^ and CFC in the GWAS analysis. The in silico analysis suggests that the associated QTL interval harbors a *putative mixed*-*linked glucan synthase* (*CesA*) involved in carbohydrate metabolism and mediation of cellulose biosynthesis and cell wall organization (Somerville 2006). Mutation in *CesA* enhances drought and osmotic stress tolerance in Arabidopsis (Zhu et al. 2010).

GWAS can identify shared mechanisms underlying multiple traits in a systematic way (Pickrell et al. 2016). The integration of genetic studies for multiple traits is considered to be a powerful approach to improve the identification of relevant genetic architectures of complex traits since pleiotropy increases the statistical power to detect genetic variants (Kim et al. 2018). The SNP marker detected at 99.04 cM (PVE = 4.4–14.7%) on 6AL is associated with *ABS*/*RC*, *DIo*/*RC*, *shoot Na*^+^ uptake and *shoot K*^+^*/Na*^+^ ratio and is proximal to ST QTL reported for *F*_m_, *F*_v_*/F*_m_ (Li et al. 2012) and *shoot Na*^+^ uptake (Genc et al. 2010). The ORFs harboring this marker is orthologous to the *OPAQUE1* gene. *OPAQUE1* is salt-responsive and is directly influenced by the vacuolar Na^+^/H^+^ antiporter (Sottosanto et al. 2007). The SNP marker (*Jagger_c1134_353*) at 140.87 cM on 6AL had a genome-wide association with *F*_v_ and *shoot Na*^+^ uptake and was located in the ORFs of *TaABCF3* transporter, suggesting that associated region may be involved in Na^+^ transport and/or plant ion homeostasis. Reports have indicated that the 6AL harbors three wheat plasma membrane Na^+^ transporters (Pearce et al. 2014). A gene homolog of *TaABCC3* was found in the Q.chl*Qu(2DS) region (at 8.52 cM) on 2DS that had a significant effect on *F*_m_*/F*_o_, *F*_v_*/F*_o_ and CFC ST traits in this study. The Q.chl*Qu(2DS) region coincided with the QTL interval previously reported for important agronomic and developmental QTL/genes including QTL *F*_m_ (Zhang et al. 2010b), *qGY2Da* (Zhang et al. 2009), *Rht8* and *Ppd*-*D1* (Pestsova and Röder 2002; Gasperini et al. 2012). The *TaABCC3* has also been implicated in detoxification, heavy-metal sequestration, chlorophyll catabolite transport, chloroplast catabolite turnover, and ion-channel regulation and has a positive effect on grain formation and mycotoxin tolerance in wheat (Wanke and Üner Kolukisaoglu 2010; Walter et al. 2015). The SNP marker at 68.45 cM on 4BL has a strong genetic effect on *shoot K*^+^*/Na*^+^ ratio, *ABS*/*RC* and *TRo/RC.* The ORFs sequence carrying this SNP marker is *NADH dehydrogenase complex* (plastoquinone) assembly (*NRAMP*-*2*), and it is involved in photosynthetic electron transport in photosystem I, metal ion transporter/homeostasis and inorganic anion transport (Kriventseva et al. 2014).

### Gene-expressional analyses

Salinity stress-induced gene-expressional changes are key components of the molecular mechanisms by which plants adapt to environmental challenges (Hirayama and Shinozaki 2010). The differential gene expression is an essential response of plants against abiotic stresses (Kumari and Pandey 2013). Twenty-eight candidate genes encompassing the SNP markers detected in this study showed differential expressions between salt-tolerant and salt-sensitive wheat genotypes, indicating that the associated regions may be linked to genes conferring salt tolerance and can represent an important resource for the improvement of wheat salt stress adaptation. Among them, *OPAQUE1*, *SPS1*, *NRAMP*-*2* and *USP* were strongly and differentially expressed. Unlike the salt-sensitive genotype, the up-regulation of these genes (relative to control) was observed in the salt-tolerant genotype. Reports have shown that *OPAQUE1* exhibits an altered transcript abundance during salinity stress (Cheng et al. 2009; Peng et al. 2009) and acts as a cytoskeleton-cell wall linker to callose synthase complexes in the plasma membrane. The activation and increased expression of *OPAQUE1* are parts of plant stress response mechanisms (Kosová et al. 2013). The expressions of *OPAQUE1*, *TaABCF3*, and *NAD(P)H* were also validated with the qRT-PCR approach using two tolerant and two sensitive wheat genotypes (Fig. 4).

The analyses of gene expression kinetics of stress treatments are important and should be considered when expression analyses are performed to identify stress-responsive genes (Deyholos 2010). In this study, the expression kinetics of 15 underlying candidate genes was analyzed at three time points under saline and non-saline conditions. Findings revealed that the gene expressions are genotype, salt stress, and stress duration dependent. Both salt-tolerant and salt-sensitive genotypes exhibited differential expression patterns across the stress durations considered, with salt-sensitive genotypes having higher transcript amount in the first 5 days under salt stress. However, the salt-tolerant genotype displayed greater gene expressions as the salt stress progressed up to 11 days. Three candidate genes including *OPAQUE1*, *hxB*, and *NRAMP*-*2* showed an earlier differential response (0–5 days), while *MPK9* was differentially expressed after 15 days under salt stress. The differential expression time points observed between the tolerant and sensitive genotypes might coincide with the period described by Munns and Tester (2008) as “ionic phase”—a period when Na^+^ toxicity (due to high concentrations of Na^+^ ion) has ensued in the plant tissues, and only plants (salt-tolerant) that are able to exclude the excess Na^+^ and/or compartmentalize Na^+^ in vacuoles (as a result of increased activities of stress-responsive genes) can adapt under the salt stress conditions.

The genetic intervals in LD with the pleiotropic SNP markers at 68.45 cM on 4BL and 90.04 cM on 6AL were scanned for other possible causative candidate genes. The expression profiles of all the genes in the associated QTL intervals of *scaffold41600* (~ 13 Mb containing 61 candidate genes) on 4BL and *scaffold126294.1 (*~ 12 Mb containing 141 candidate genes) on 6AL revealed that *TraesCS4B01G254300.1* (*NRAMP*-*2)* and *TraesCS6A01G336500.1* (*OPAQUE1*) genes showed highest differential expressions between the salt-tolerant and salt-sensitive wheat genotypes after 24 days under salt stress. This result supports the GWAS results and provides strong evidence that the QTL identified on 4BL and 6AL may be harboring salt stress-responsive genes and should be considered for gene-based marker development for salt stress tolerance in wheat; not only because the genes that are linked to the associated pleiotropic markers are showing the highest differential expression between the salt-tolerant and salt-sensitive genotypes but because the biological functions of these genes are related and relevant to the traits evaluated. However, the involvement of both genes in the salt stress response pathways and salt tolerance in wheat need to be validated functionally before exploitation in the development of functional markers for salt stress tolerance.

### Analyses of *NRAMP-2*, *TaABCF3* and *CSLF1* promoter regions

Transcription factors (TFs) play major roles in the regulation of genes involved in development and tolerance to biotic and abiotic stresses (Dang et al. 2012), by serving as nuclear effectors of multiple signaling cascades and often involved in posttranslational modifications (Tootle and Rebay 2005). The analyses of the promoter are crucial for improving the basic understanding of the expressions of adaptive/stress-responsive genes and will promote their application in plant breeding. Variation in the TF will affect more than one downstream modification as TFs are hierarchically higher and have the ability to regulate many downstream targets (Poser and Bosserhoff 2004), giving rise to gradations of gene expression. In this study, the promoter regions of *NRAMP*-*2*, *TaABCF3* and *CSLF1* were also analyzed for possible detection of ST motifs/cis-regulatory elements in two salt-tolerant and two salt-sensitive wheat genotypes.

The two salt-tolerant genotypes contain unique DNA-binding domains *TF_motif_seq_0239****/****Dof* and *PBF* at 99-bp upstream of the *NRAMP*-*2* translation start codon, whereas these TF features were lacking and/or replaced by the broad-complex 3 and/or hunchback DNA-binding domains in the two salt-sensitive genotypes, indicating that the presence of the *Dof*-*/PBF*-motifs may have contributed to the up-regulation of the *NRAMP*-*2* transcriptions in the tolerant genotypes *vis*-*à*-*vis* the sensitive ones. This result provides an initial indication that these motifs may be regulating salt stress response in *NRAMP*-*2*, but further validation via reporter gene experiments is needed. Yanagisawa (2000) reported that the Dof proteins are unique to plants and play regulatory roles in the expression of multiple genes involved in pathways for carbon metabolism in maize. The *NRAMP*-*2* gene is involved in the non-photochemical reduction of plastoquinones, cyclic electron transport around photosystem as well as chlororespiration in the thylakoids (Rumeau et al. 2005). In this study, the SNP marker linked to *NRAMP*-*2* had a significant genome-wide association with the specific energy fluxes per reaction center for absorption (*ABS*/*RC*) and dissipation-(*DIo*/*RC*), and the *shoot K*^+^*/Na*^+^ ratio, a strong indication that the detected marker could be an eQTL and may serve as a valuable genetic resource for ST improvement in wheat.

The regulatory motif variation in *CSLF1* gene was genotype-specific. Genotype *UZ*-*11CWA*-*08* contains the homeodomain motif family—*bZIP*, *HD*-*ZIP* and *GATA*-binding proteins (at 581 and 554 bp, respectively) upstream of the translation start codon of the gene, but these motifs were lacking in remaining genotypes. Homeodomain motifs act as growth regulators in response to water deficit, whereas the *GATA*-binding proteins are associated with light-regulated and tissue-specific response in plants (Teakle et al. 2002; Chow et al. 2015). The linked markers for *CSLF1* were found to be genetically associated with *F*_v_*/F*_m_ (Maximum quantum yield of PSII). On the other hand, the salt-sensitive genotype *UZ*-*11CWA*-*24* contains the *SPI*-*B* and *NF*-*Y* motifs at position 618-bp and 540-bp upstream of the translation start codon, respectively. The report by Ogra et al. (2001) indicated that the *SPI* motifs regulate the salt stress-dependent posttranscriptional stabilization and have been implicated as a negative regulator of transcription mediated by specific metal-responsive elements. A genome-wide expression analysis has also revealed that the *NFY* motifs are important plant regulators of abiotic stress tolerance (Petroni et al. 2012; Laloum et al. 2013).

### Detection of polymorphisms in the associated gene coding regions

Changes in DNA sequence of the coding genes can cause alterations in the proteins (Studer et al. 2013), and studies aimed at analyzing how these alterations affect protein functions are essential for gaining an in-depth understanding of stress adaptation. The alignment of the expressed sequence tags of *OPAQUE1* and *NRAMP*-*2* genes revealed several amino acid substitution sites between *Altay2000* and *Bobur*. For instance, *OPAQUE1* (*Traes_6AL_891456790.1*) had four substitutions: C1529G, A1549V, R1626G and C1628W, based on single nucleotide polymorphisms, at exon 37 that had a significant genome-wide association with multiple ST traits. The R/G substitution is the most non-conservative modified variation, indicating that it may have contributed to the alteration in the gene function during salt stress by replacing the polar positively charged arginine with tiny apolar hydrophobic glycine. However, further studies are very essential to validate the functional consequences of the substitution sites identified in this study in salt stress adaptation.

#### Author contribution statement

FCO and JL conceived and acquired the project research grant; AB BCO and RCS planned all the research and experiments; BCO designed all the experiments and analyzed/interpreted the data; AB and JL supervised all the experiments; BCO prepared the manuscript; AB, FCO, JL, MB and RCS reviewed the manuscript; and all authors approved the manuscript.

## Electronic supplementary material

Below is the link to the electronic supplementary material.

Supplementary material 1 (DOCX 977 kb)

Supplementary material 2 (DOCX 78 kb)
