# Supplementary material for: Genetic and transcriptional variations in NRAMP-2 and OPAQUE1 genes are associated with salt stress response in wheat
Source: Theor Appl Genet. 2018 Nov 3;132(2):323–46. doi: 10.1007/s00122-018-3220-5 (PMC6349800; doi:10.1007/s00122-018-3220-5)
Supplement: Supplementary file 1 — Supplementary material 1 (DOCX 977 kb) [file 122_2018_3220_MOESM1_ESM.docx]

**SUPPLIMENTARY FIGURES**


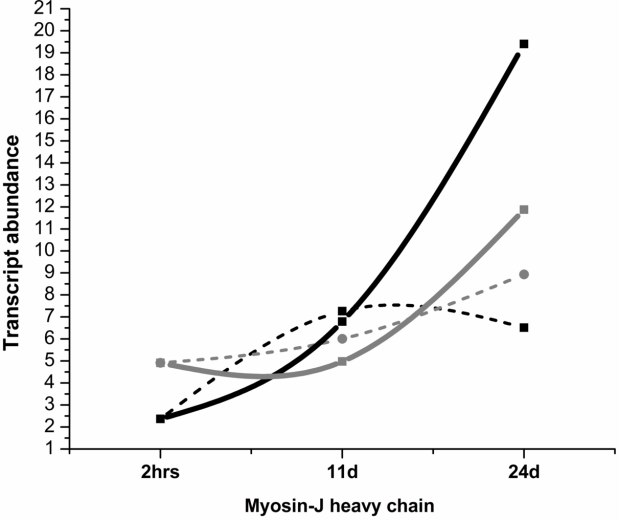

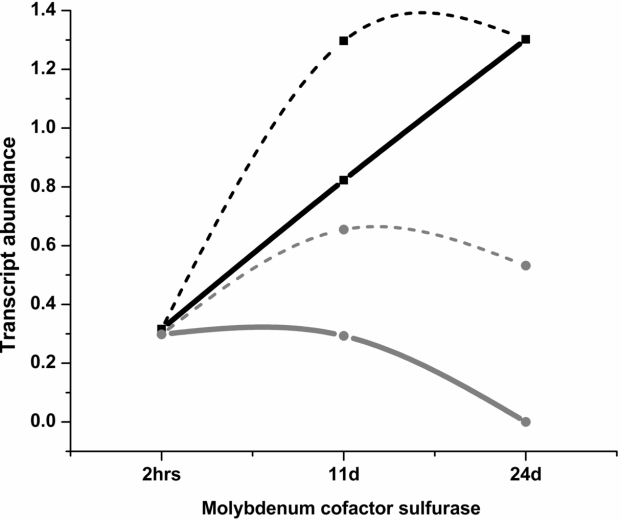

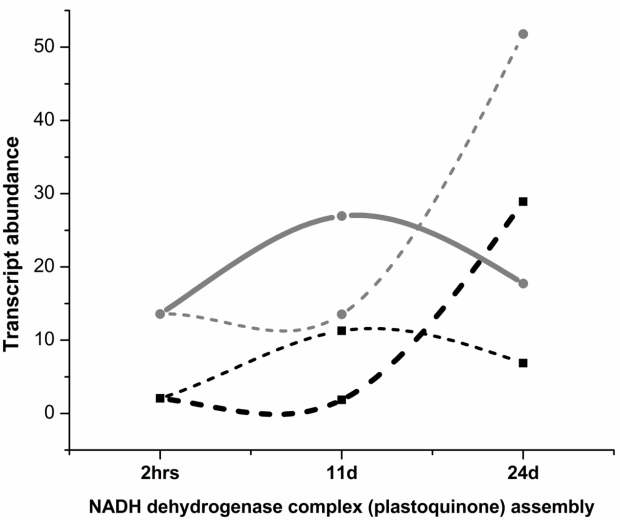

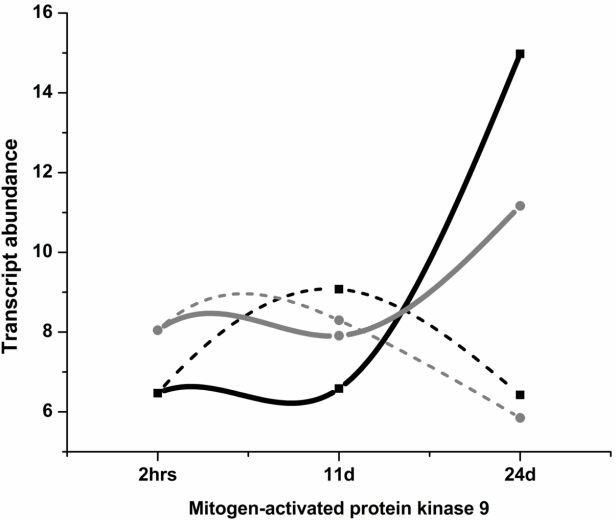

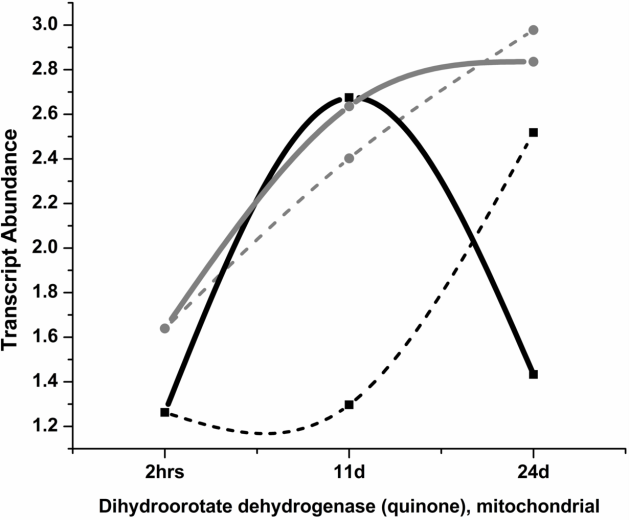

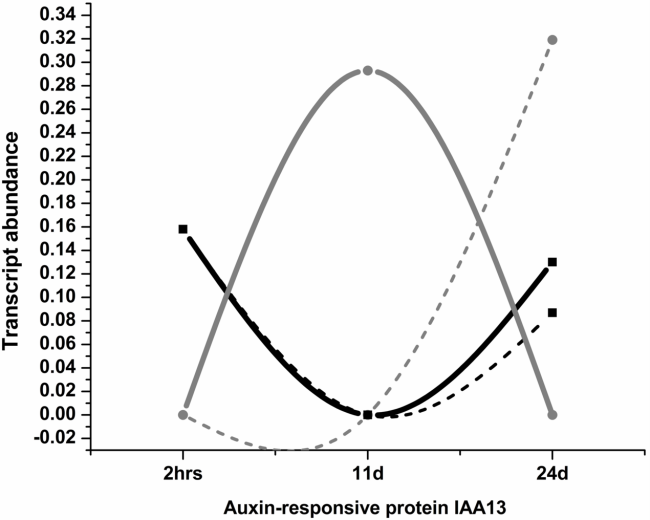


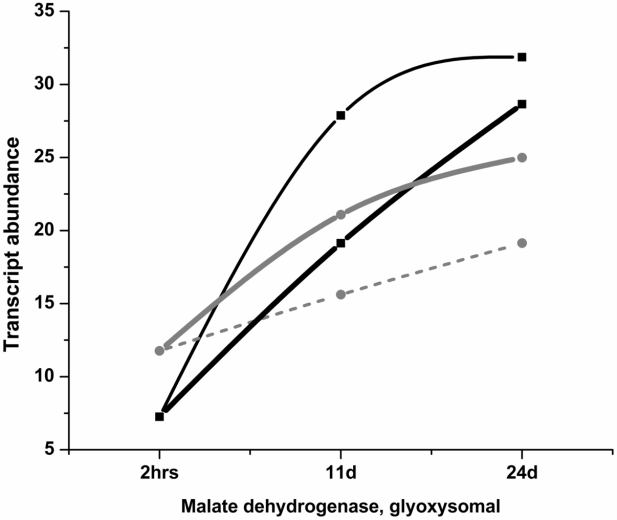

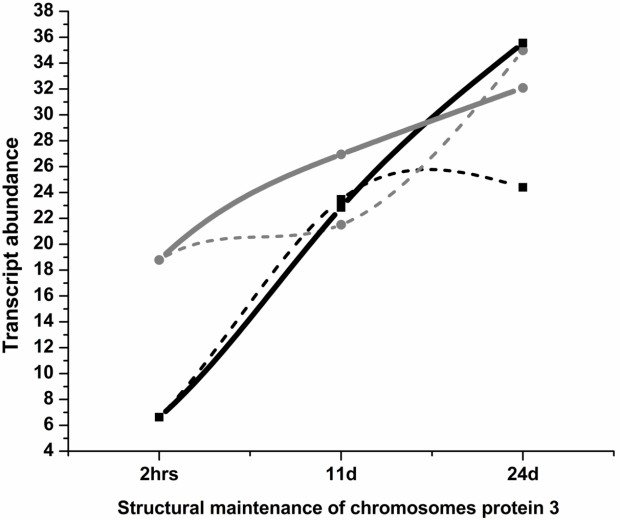

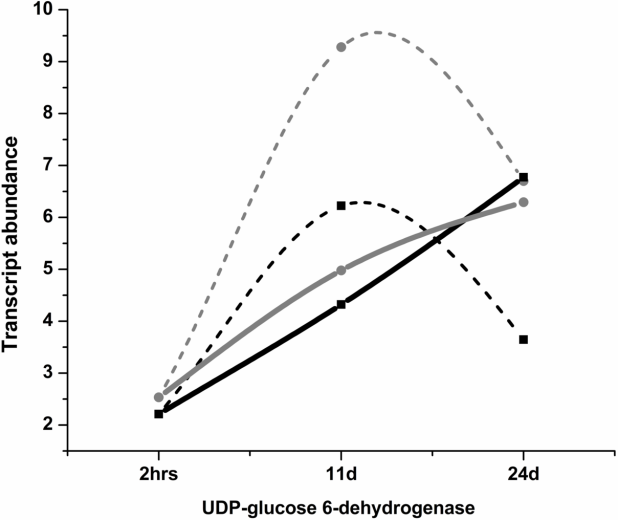

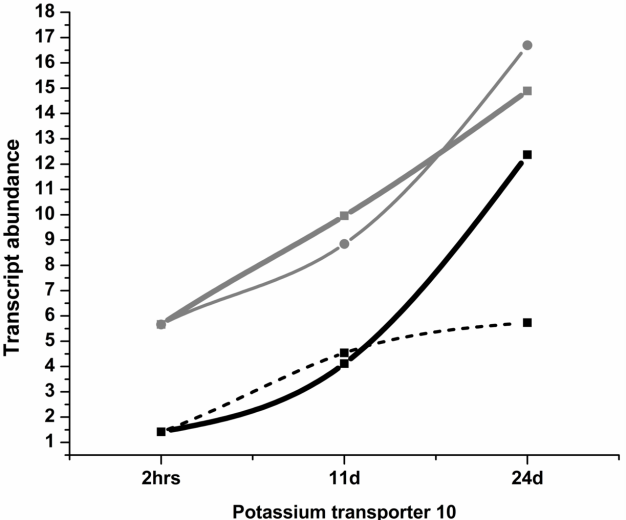

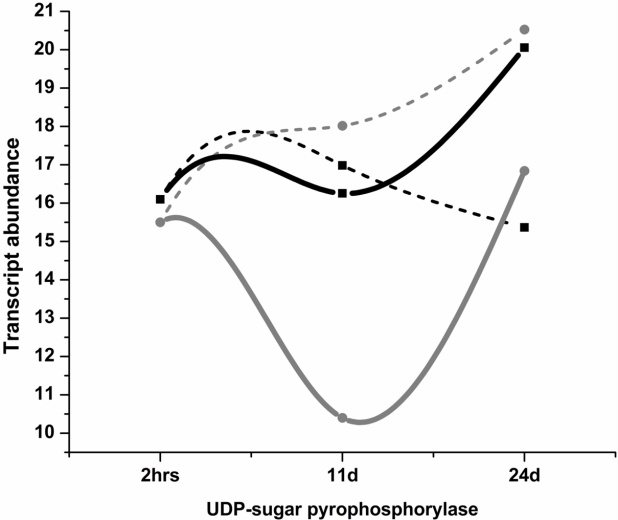

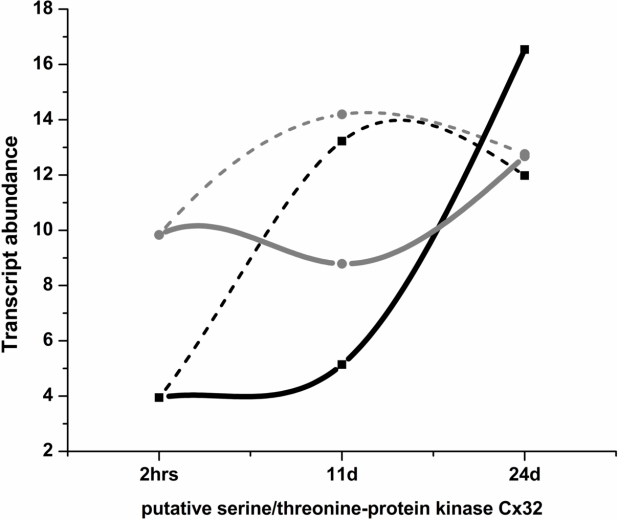


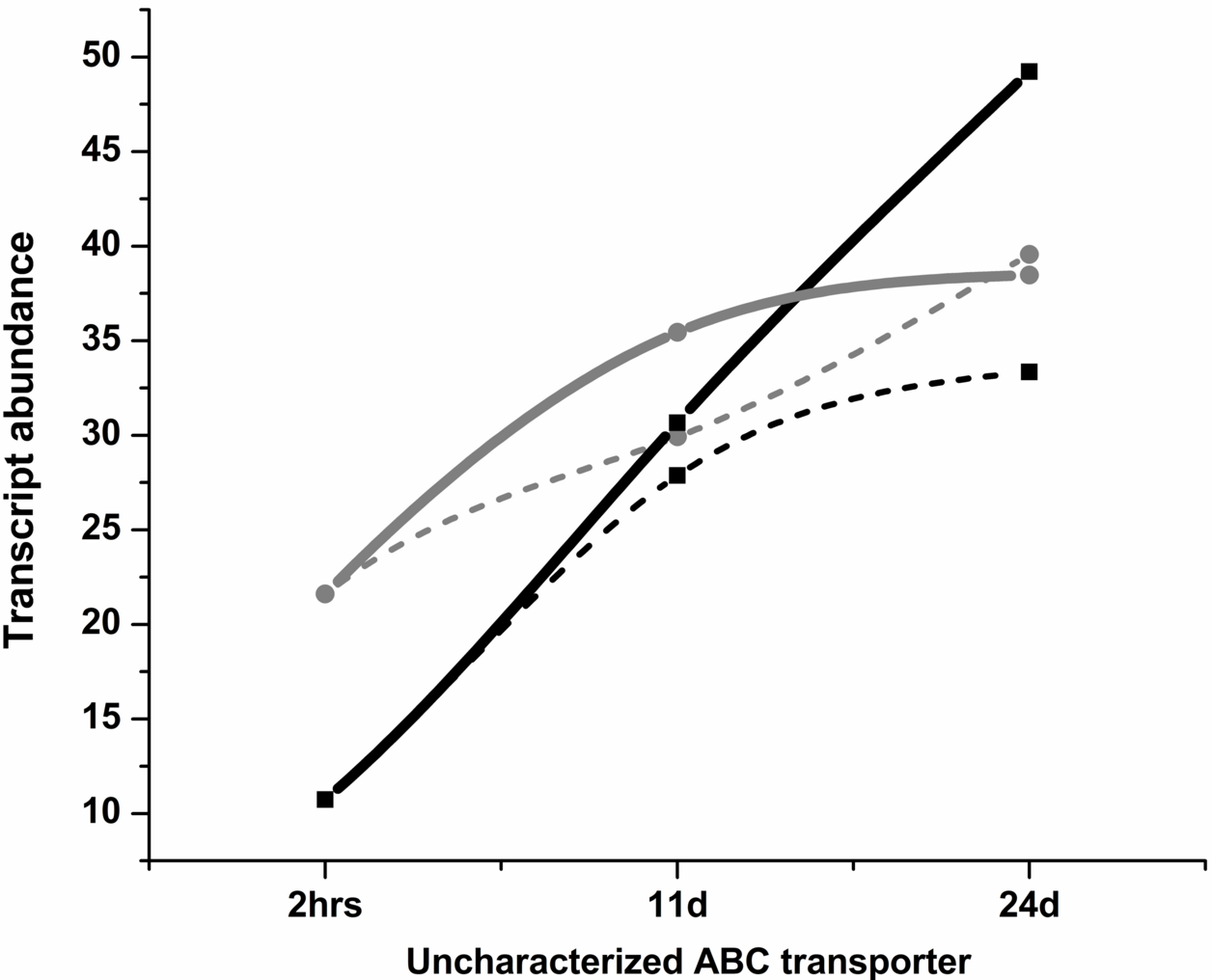

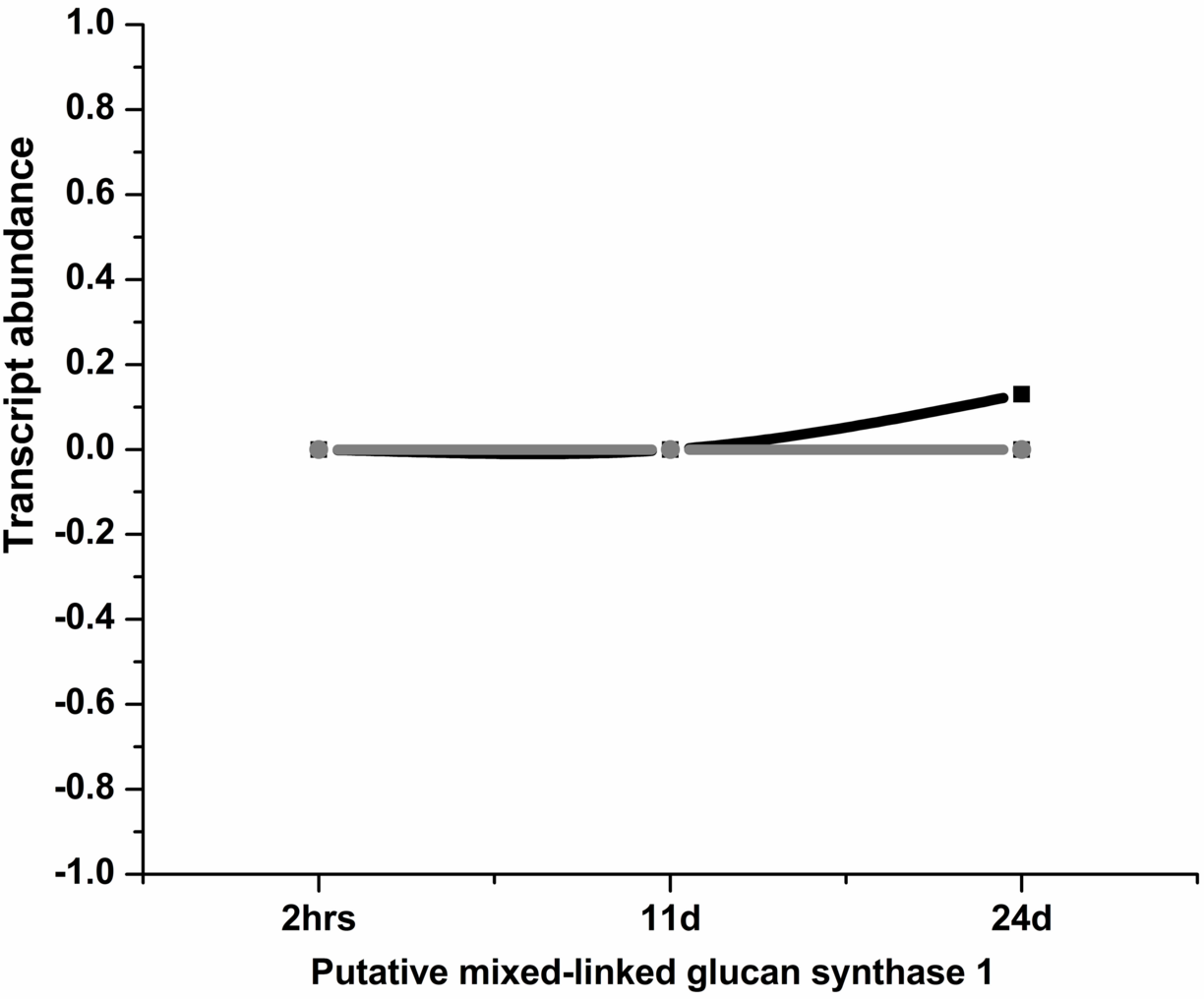

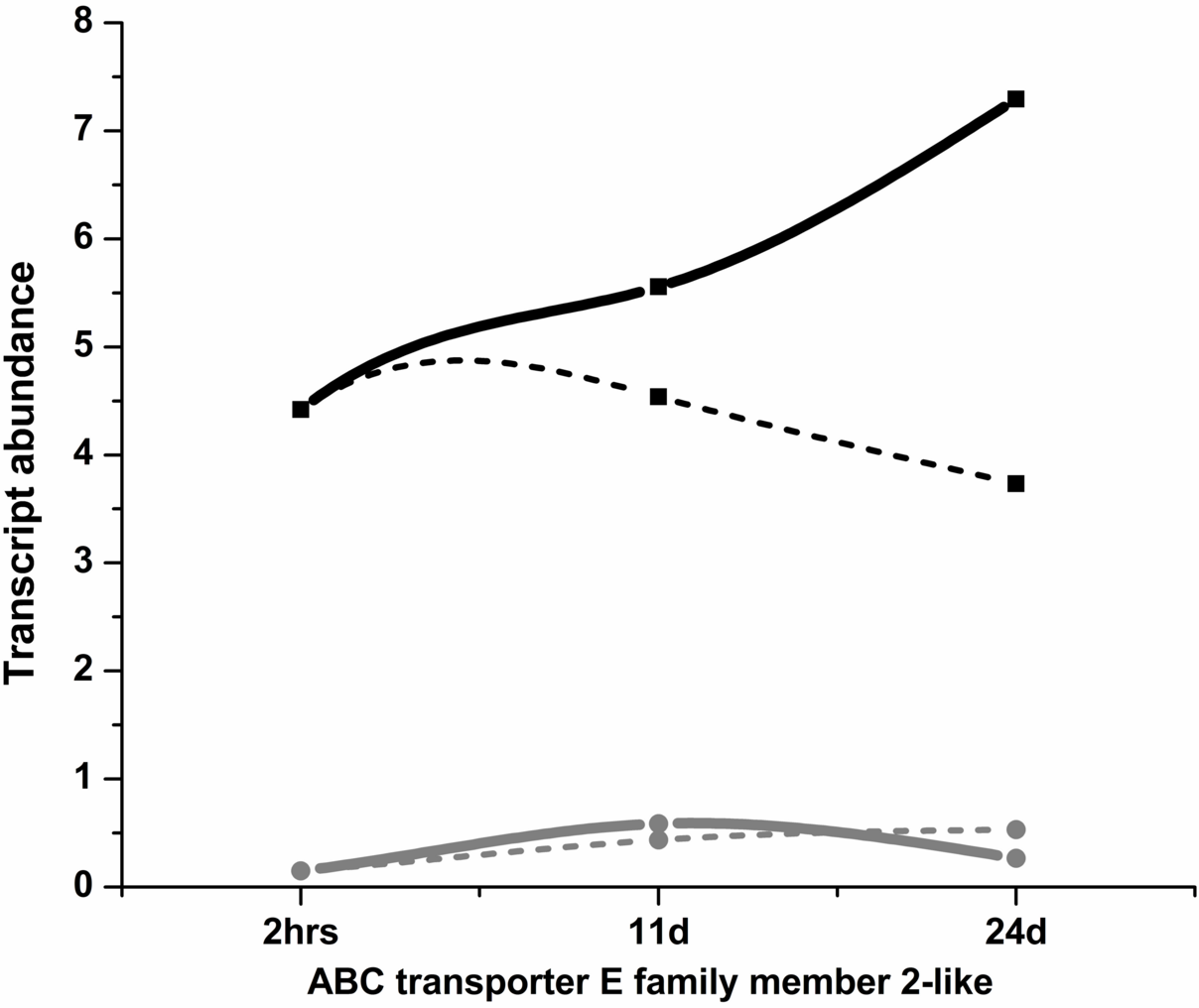


**Figure S1** MACE analysis showing the expression kinetics of the associated salt tolerance genes over a period of 24d in salt tolerant (in black) and salt sensitive (in grey) wheat genotypes. The “**thick**” and “**dotted**” lines indicate the gene expression kinetics over-time in saline and non-saline conditions, respectively.

Atlay2000 --TTCCGGGGCGTTGCTTGAAGCAACTTTACTCCATGGCGACTCTTCTGGATTTTTCCGAGTTTCGTTCAAAAGGAGGAGACAGATACAG

UZ-11CWA-8 --TTCCGGGGCGTTGCTTGAAGCAACTTTACTCCATGGCGACTCTTCTGGATTTTTCCGAGTTTCGTTCAAAAGGAGGAGACAGATACAG

UZ-11CWA-24 --TTCCGGGGCGTTGCTTGAAGCAACTTTACTCCATGGCGACTCTTCTGGATTTTTCCGAGTTTCGTTCAAAAGGAGGAGACAGATACAG

Bobur --TTCCGGGGCGTTGCTTGAAGCAACTTTACTCCATGGCGACTCTTCTGGATTTTTCCGAGTTTCGTTCAAAAGGAGGAGACAGATACAG

****************************************************************************************

Atlay2000 TAGATCTTTATCGCAGTATTCTCATTTTATTTTTTTGATTGATTGATGCCGGACGTGCAAAAAGGCAGTTAATGTTGGATTAGGAAATGG

UZ-11CWA-8 TAGATCTTTATCGCAGTATTCTCATTTTATTTTTTTGATTGATTGATGCCGGACGTGCAAAAAGGCAGTTAATGTTGGATTAGGAAATGG

UZ-11CWA-24 TAGATCTTTATCGCAGTATTCTCATTTTATTTTTTTGATTGATTGATGCCGGACGTGCAAAAAGGCAGTTAATGTTGGATTAGGAAATGG

BUBUR TAGATCTTTATCGCAGTATTCTCATTTTATTTTTTTGATTGATTGATGCCGGACGTGCAAAAAGGCAGTTAATGTTGGATTAGGAAATGG

******************************************************************************************

Atlay2000 AGCTATAAATTCGTTCCAACA**AAAGC**CAGTCCTGATTTCACGAAGGAGTTAGAGGAACTCCCATCCCCCGACACACTAGCCCCTCAACCC

UZ-11CWA-8 AGCTATAAATTCGTTCCAACAAAAGCCAGTCCTGATTTCACGAAGGAGTTAGAGGAACTCCCATCCCCCGACACACTAGCCCCTCAACCC

UZ-11CWA-24 AGCTATAAATTCGTTTCAACAAAAACCAGTCCTGATTTCACGAAGGAGTTAGAGGAACTCCCATCCCCCGACACACTAGCCCCTCAACCC

Bobur AGCTATAAATTCGTTTCAACAAAAACCAGTCCTGATTTCACGAAGGAGTTAGAGGAACTCCCATCCCCCGACACACTAGCCCCTCAACCC

***************.********.*****************************************************************

ATLAY2000 ACTCTCCACTCGCGCACGCATCCTCCTCCGCTCCC**ATGGCGTCCCGCGACCTCGCCGAGAGCCTGCTCCCCGGCGGGGGCGCCTCCGCCT**

UZ-11CWA-8 ACTCTCCACTCGCGCACGCATCCTCCTCCGCTCCC**ATGGCGTCCCGCGACCTCGCCGAGAGCCTGCTCCCCGGCGGGGGCGCCTCCGCCT**

UZ-11CWA-24 ACTCTCCACTCGCGCACGCATCCTCCTCCGCTCCC**ATGGCGTCCCGCGACCTCGCCGAGAGCCTGCTCCCCGGCGGGGGCGCCTCCGCCT**

Bobur ACTCTCCACTCGCGCACGCATCCTCCTCCGCTCCC**ATGGCGTCCCGCGACCTCGCCGAGAGCCTGCTCCCCGGCGGGGGCGCCTCCGCCT**

******************************************************************************************

Atlay2000 **GCCTC---CTCCTCCCACGACGAGTACGAGGAGCGCGCGTACGACTCGGACGACAAGGTCTCCATCTCCA--**

UZ-11CWA-8 **GCCTC---CTCCTCCCACGACGAGTACGAGGAGCGCGCGTACGACTCGGACGACAAGGTCTCCATCTCCA--**

UZ-11CWA-24 **GCCTC---CTCCTCCCACGACGAGTACGAGGAGCGCGCGTACGACTCGGACGACAAGGTCTCCATCTCCA--**

Bobur **GCCTCTCTCTCCTCCCACGACGAGTACGAGGAGCGCGCGTACGACTCGGACGACAAGGTCTCCATCTCCA--**

***** **************************************************************

**Figure S2** Nucleotide sequence comparisons of the sequenced 5′- promoter region of **the** *NADH dehydrogenase complex* (plastoquinone) assembly (Metal transporter *NRAMP-2*) in salt-tolerant (***Atlay2000* and *UZ-11CWA-8*) and** salt-sensitive (***UZ-11CWA-24 and Bobur*)** wheat genotypes, showing the presence of putative *Dof*- 5'-AA[AG]G-3' and PBF- 5′-AAAGC-3′ motifs (in pink) in the tolerant wheat genotypes and presence of *broad*-*complex 3*- 5′-TCAACAAAAAC-3′)- and *hunchback*- 5′-CAACAAAAAC-3′ motifs (in green) in the sensitive genotypes. The grey and underlined sequence region correspond to the gene coding region from the start codon. The coding region of Bobor contains additional nucleotides (in red) that was not present in the coding regions of the remaining three wheat genotypes.

Altlay2000 --GATTGGATCTTCTAGGGCCTCCAGCTCACGCGCCGTGCATGCACTCTCGCAGTCACAGCGCAGTCATAGGGTAGGGAGTAGTAGATCGGA

UZ-11CWA-8 --GATTGGATCTTCTAGGGCCTCCAGCTCACGCGCCGTGCATGCACTCTCGCAGTCACAGCGCAATCATAGGGTAGGGAGTAGTATATCGGA

UZ-11CWA-24 --GATTGGATCTTCTAGGGCCTCCAGCTTCCGCTCCGTGCACGCACTCTCGCAGTCACAGCGCATTCATAGGGTAAGGGGTGGTAGATCGGA

Bobur --GATTGGATCTTCTAGGGCCTCCAGCTCACGCGCCGTGCATGCACTCTCGCAGTCACAGCGCAGTCATAGGGTAGGGAGTAGTAGATCGGA

**************************. *** *******.********************** **********.**.**.*** ******

Altlay2000 TCGTT-GAACCAATGCATGCACATGCAGGCTGGGGCCGGGCGACGCAGGCAAAACAAATATGCCTGACCTTCCGCGCAGGCGCAGCTACT

UZ-11CWA-8 TCGATAGAACCAATGCATGCACATGCAGGCTGGGGCCGGGCGACGCAGGCAAAACAAATATGCCTGACCTTCCGCGCAGGCGCAGCTACT

UZ-11CWA-24 TCGAT-GAACCAATGCATGCACATGCAGGCTGGGGCCGGGCGACGCAGGCAAAACAAATATGCCTGACCTTCCGCGCAGGCGCAGCTACT

Bobur TCGTT-GAACCAATGCATGCACATGCAGGCTGGGGCCGGGCGACGCAGGCAAAACAAATATGCCTGACCTTCCGCGCAGGCGCAGCTACT

*** * ************************************************************************************

Altlay2000 AAACTAACTGTGCTTGCTTGACCAAGTGGGCAGTGGCCAGTGCAACCGCCGGAAATGCCTGACACGACGCGGAATAATGCGGGGCACGCA

UZ-11CWA-8 AAACTAACTGTGCTTGCTTGACCAAGTGGGCAGTGGCCAGTGCAACCGCCGGAAATGCCTGACACGACGCGGAATAATGCGGGGCACGCA

UZ-11CWA-24 AAACTAACTGTGCTTGCTTGACCAAGTGGGCAGTGGCCAGTGCAACCGCCGGAAATGCCTGACACGACGCGGAATAATGCGGGGCACGCA

Bobur AAACTAACTGTGCTTGCTTGACCAAGTGGGCAGTGGCCAGTGCAACCGCCGGAAATGCCTGACACGACGCGGAATAATGCGGGGCACGCA

******************************************************************************************

Altlay2000 CTTGCACTTCCCACGACGACGTGCGTGCGTGCATCTTCCTTCCCGTCCCGTGGATCCCGCCGGCCCTGCACATGGAAGCCTCCACGTTTT

UZ-11CWA-8 CTTGCACTTCCCACGACGACGTGCGTGCGTGCATCTTCCTTCCCGTCCCGTGGATCCCGCCGGCCCTGCACATGGAAGCCTCCACGTTTT

UZ-11CWA-24 CTTGCACTTCCCACGACGACGTGCGTGCGTGCATCTTCCTTCCCGTCCCGTGGATCCCGCCGGCCCTGCACATGGAAGCCTCCACGTTTT

Bobur CTTGCACTTCCCACGACGACGTGCGTGCGTGCATCTTCCTTCCCGTCCCGTGGATCCCGCCGGCCCTGCACATGGAAGCCTCCACGTTTT

******************************************************************************************

Altlay2000 CTACATGGATGGCTACTTGGCTTCTATCCTAAGTGAGGACAGAGCAGACCACCGATCGTTCGGAATCACAGATCTCGAGCGGATTCCTTC

UZ-11CWA-8 CTACATGGATGGCTACTTGGCTTCTATCCTAAGTGAGGACAGAGCAGACCACCGATCGTTCGGAATCACAGATCTCGAGCGGATTCCTTC

UZ-11CWA-24 CTACATGGATGGCTACTTGGCTTCTATCCTAAGTGAGGACAGAGCAGACCACCGATCGTTCGGAATCACAGATCTCGAGCGGATTCCTTC

Bobur CTACATGGATGGCTACTTGGCTTCTATCCTAAGTGAGGACAGAGCAGACCACCGATCGTTCGGAATCACAGATCTCGAGCGGATTCCTTC

******************************************************************************************

Altlay2000 TGGTCTCTGTGCTTCGCCCTTGCCCAAGCCCAACGATCGAGGCCGTCCTAGTACCTCGCCGTTCCTCCGACTCTTCCTGCCTCTCTATAT

UZ-11CWA-8 TGGTCTCTGTGCTTCGCCCTTGCCCAAGCCCAACGATCGAGGCCGTCCTAGTACCTCGCCGTTCCTCCGACTCTTCCTGCCTCTCTATAT

UZ-11CWA-24 TGGTCTCTGTGCTTCGCCCTTGCCCAAGCCCAACGATCGAGGCCGTCCTAGTACCTCGCCGTTCCTCCGACTCTTCCTGCCTCTCTATAT

Bobur TGGTCTCTGTGCTTCGCCCTTGCCCAAGCCCAACGATCGAGGCCGTCCTAGTACCTCGCCGTTCCTCCGACTCTTCCTGCCTCTCTATAT

******************************************************************************************

Altlay2000 AAACCCTGCGCCTCACTTGCTCACCTCGTGCACCACTGGCTGGCTGGCTGGCTCGAGTTGTAGCTTTTCCTCCCTTCCAGCTCCGGTCGG

UZ-11CWA-8 AAACCCTGCGCCTCACTTGCTCACCTCGTGCACCACTGGCTGGCTGGCTGGCTCGAGTTGTAGCTTTTCCTCCCTTCCAGCTCCGGTCGG

UZ-11CWA-24 AAACCCTGCGCCTCACTTGCTCACCTCGTGCACCACTGGCTGGCTGGCTGGCTCGAGTTGTAGCTTTTCCTCCCTTCCAGCTCCGGTCGG

Bobur AAACCCTGCGCCTCACTTGCTCACCTCGTGCACCACTGGCTGGCTGGCTGGCTCGAGTTGTAGCTTTTCCTCCCTTCCAGCTCCGGTCGG

******************************************************************************************

Altlay2000 GTTAGCTCGGACTGGGAGACATGGCGTCGGCGGTCGGTGCTGGTGGGGCAAATGCCGGCCTCGCCGACCCGCTGCTGGCGAGCCGCGACG

UZ-11CWA-8 GTTAGCTCGGACTGGGAGACATGGCGTCGGCGGTCGGTGCTGGTGGGGCAAATGCCGGCCTCGCCGACCCGCTGCTGGCGAGCCGCGACG

UZ-11CWA-24 GTTAGCTCGGACTGGGAGACATGGCGTCGGCGGTCGGTGCTGGTGGGGCAAATGCCGGCCTCGCCGACCCGCTGCTGGCGAGCCGCGACG

Bobur GTTAGCTCGGACTGGGAGACATGGCGTCGGCGGTCGGTGCTGGTGGGGCAAATGCCGGCCTCGCCGACCCGCTGCTGGCGAGCCGCGACG

******************************************************************************************

Altlay2000 GCGGTGCCAAGAAGCCGGTCGGCGCCAAGGGCAAGCACTGGGTGGCCGCCGACAAAGACCAGCGCCGGGCCGCCAAGGAGAGCGGCGGCA

UZ-11CWA-8 GCGGTGCCAAGAAGCCGGTCGGCGCCAAGGGCAAGCACTGGGTGGCCGCCGACAAAGACCAGCGCCGGGCCGCCAAGGAGAGCGGCGGCG

UZ-11CWA-24 GCGGTGCCAAGAAGCCGGTCGGCGCCAAGGGCAAGCACTGGGTGGCCGCCGACAAAGACCAGCGCCGGGCCGCCAAGGAGAGCGGCGGCG

Bobur GCGGTGCCAAGAAGCCGGTCGGCGCCAAGGGCAAGCACTGGGTGGCCGCCGACAAAGACCAGCGCCGGGCCGCCAAGGAGAGCGGCGGCG

*****************************************************************************************.

Altay2000 AGGAGGGCAGGCCGTTGCTGTTCCGGACGTACAAGGTCAAAGGCACCCTCCTGCATCCCTACAGGTAAGCGCGCCAAATCCAAGCGCGTT--

UZ-11CWA-8 AGGAGGGCAGGCCGTTGCTGTTCCGGACGTACAAGGTCAAAGGCACCCTCCTGCATCCCTACAGGTAAGCGCGCCAAATCCAAGCGCGTT--

UZ-11CWA-24 AGGAGGGCAGGCCGTTGCTGTTCCGGACGTACAAGGTCAAAGGCACCCTCCTGCATCCCTACAGGTAAGCGCGCCAAATCCAAGCGCGTT--

Bobur AGGAGGGCAGGCCGTTGCTGTTCCGGACGTACAAGGTCAAAGGCACCCTCCTGCATCCCTACAGGTAAGCGCGCCAAATCCAAGCGCGTT--

******************************************************************************************

**Figure S3** Nucleotide sequence comparisons of the sequenced 5′- promoter region of **p***utative mixed-linked glucan synthase 1* in salt-tolerant (**Atlay2000 and UZ-11CWA-8) and** salt-sensitive (**UZ-11CWA-24 and Bobur)** wheat genotypes, showing homeodomain motif - *bZIP; HD-ZIP*- (5′-[GCAATCATAG](https://biowulf.bu.edu/cgi-bin/clover/motifviz.cgi#seq160)-3), GATA- (5′-[GATA](https://biowulf.bu.edu/cgi-bin/clover/motifviz.cgi#seq160)-3) (in red), SPI-B- (5′-TTCCGCT-3′) and NF-Y (5′-ATGAACCAATGCATGC-3′) (in green) DNA binding proteins. The grey and underlined sequence region corresponds to the gene coding region from the start codon.

UZ-11CWA-08 –-TTCGAGTTCCTTCGCCGATTG-ACTTTAGTTGAGCCAG-CCAGTAGCGTCATCAAGCGCTCGTATACTAAACCTGCGCTGAGAGCGCCGG

UZ-11CWA-24 --TTCGAGTTCCTTCGCCGATTGAACTTTAGTTGAGCCAGCCCAGTAGCGTCATCAAGCGCTCGTATACTAAACCTGCGCTGAGAGCGCCGG

Bobur --TTCGAGTTCCTTCGCCGATTGAACTTTAGTTGAGCCAGCCCAGTAGCGTCATCAAGCGCTCGTATACTAAACCTGCGCTGAGAGCGCCGG

********************* **************** ***************************************************

UZ-11CWA-08 TTAGGAGCTCCCGCAAAAACCCTCGTCGAGGCACCCAAAATATTTCCACTTCCTTTCTCGGCTAAAAGCCCAACAATGCCCTGTCTCCCG

UZ-11CWA-24 TTAGGAGCTCCCGCAAAAACCCTCGTCGAGGCACCCAAAATATTTCCACTTCCTTTCTCGGCTAAAAGCCCAACAATGCCCTGTCTCCCG

Bobur TTAGGAGCTCCCGCAAAAACCCTCGTCGAGGCACCCAAAATATTTCCACTTCCTTTCTCGGCTAAAAGCCCAACAATGCCCTGTCTCCCG

******************************************************************************************

UZ-11CWA-08 TTTTTTCAAACACACCATTAAAATTGATCTTTATGTATGGTGGAATCATTTGCGCCGGCGCACGGGAGGCAATTGAGAATTCATCCGGTT

UZ-11CWA-24 TTTTTTCAAACACACCATTAAAATTGATCTTTATGTATGGTGGAATCATTTGCGCCGGCGCACGGGAGGCAATTGAGAATTCATCCGGTT

Bobur TTTTTTCAAACACACCATTAAAATTGATCTTTATGTATGGTGGAATCATTTGCGCCGGCGCACGGGAGGCAATTGAGAATTCATCCGGTT

******************************************************************************************

UZ-11CWA-08 CAACTCCTCAAGGAATGTTAATGCAAAAGAAAAGAACAGATAACGGGATTTGGTACTTATTCTCATGAATAATCTTCCGACGAGCTTTAA

UZ-11CWA-24 CAACTCCTCAAGGAATGTTAATGCAAAAGAAAAGAACAGATAACGGGATTTGGTACTTATTCTCATGAATAATCTTCCGACGAGCTTTAA

Bobur CAACTCCTCAAGGAATGTTAATGCAAAAGAAAAGAACAGATAACGGGATTTGGTACTTATTCTCATGAATAATCTTCCGACGAGCTTTAA

******************************************************************************************

UZ-11CWA-08 AAAACATACACTATATGAGATTCATTAAAAAAAACTCTATTTTTTAGTGGGCCACGCCAAATGCTGCCGAAGGAAAAAACACTTCATCCG

UZ-11CWA-24 AAAACATACACTATATGAGATTCATTAAAAAAAACTCTATTTTTTAGTGGGCCACGCCAAATGCTGCCGAAGGAAAAAACACTTCATCCG

Bobur AAAACATACACTATATGAGATTCATTAAAAAAAACTCTATTTTTTAGTGGGCCACGCCAAATGCTGCCGAAGGAAAAAACACTTCATCCG

******************************************************************************************

UZ-11CWA-08 TTCAAGCCCTTGTGTGTGTTTTGCGTCTGAAACTGGACCTTGCGACGGGCCTCCATACGCGGAATCCTGACGATCCACGAACCCGAAGGC

UZ-11CWA-24 TTCAAGCCCTTGTGTGTGTTTTGCGTCTGAAACTGGACCTTGCGACGGGCCTCCATACGCGGAATCCTGACGATCCACGAACCCGAAGGC

BOBUR TTCAAGCCCTTGTGTGTGTTTTGCGTCTGAAACTGGACCTTGCGACGGGCCTCCATACGCGGAATCCTGACGATCCACGAACCCGAAGGC

******************************************************************************************

UZ-11CWA-08 CCATCCATCCATTGGATTGGACGAGCTGCTGCATTCGGCAGGCTAAACCCAAGCCGGCAGCCCGCGAGGTCGCACCACCGTCTCCGACGT

UZ-11CWA-24 CCATCCATCCATTGGATTGGACGAGCTGCTGCATTCGGCAGGCTAAACCCAAGCCGGCAGCCCGCGAGGTCGCACCACCGTCTCCGACGT

Bobur CCATCCATCCATTGGATTGGACGAGCTGCTGCATTCGGCAGGCTAAACCCAAGCCGGCAGCCCGCGAGGTCGCACCACCGTCTCCGACGT

******************************************************************************************

UZ-11CWA-08 CCGACCGACCCGAGCCGAGCGACGAGTAGGGTTTGCCTCGCCGCCGCCGCCGCTCGACGCACACGCGATGGCGGCGCTGGTGGAGACGAG

UZ-11CWA-24 CCGACCGACCCGAGCCGAGCGACGAGTAGGGTTTGCCTCGCCGCCGCCGCCGCTCGACGCACACGCGAGCGCGGCGCTGGTGGAGACGAG

Bobur CCGACCGACCCGAGCCGAGCGACGAGTAGGGTTTGCCTCGCCGCCGCCGCCGCTCGACGCACACGCGATGGCGGCGCTGGTGGAGACGAG

******************************************************************** ********************

UZ-11CWA-08 CATGGGGGTGGTGCGGGAGGTGCTGGG--

UZ-11CWA-24 CATGGGGGTGGTGCGGGAGGTGCTGGG--

BOBUR CATGGGGGTGGTGCGGGAGGTGCTGGG--

***************************

**Figure S4** Nucleotide sequence comparisons of the sequenced 5′- promoter region of the uncharacterized ABC transporter ATP-binding protein in salt-tolerant (**UZ-11CWA-08) and** salt-sensitive (***UZ-11CWA-24* and *Bobur*)** wheat genotypes, showing present of *TF_motif_seq_0239/DOf3* (5′-[GACTTT](https://biowulf.bu.edu/cgi-bin/clover/motifviz.cgi#seq021)-3′) and *TF_motif_seq_0263/ SORLIP1* (5′- GCCAG -3′) (in red) DNA-binding domains of *UZ-11CWA-08* and an ATG to AGC substitution (in green) that resulted in 24 bp downstream shift of the translation initiator site (in yellow) of *UZ-11CWA-24*. The grey and underlined sequence region correspond to the gene coding region from the start codon.
