# Supplementary material for: Genetic and transcriptional variations in NRAMP-2 and OPAQUE1 genes are associated with salt stress response in wheat
Source: Theor Appl Genet. 2018 Nov 3;132(2):323–46. doi: 10.1007/s00122-018-3220-5 (PMC6349800; doi:10.1007/s00122-018-3220-5)
Supplement: Supplementary file 2 — Supplementary material 2 (DOCX 78 kb) [file 122_2018_3220_MOESM2_ESM.docx]

**SUPLIMENTARY TABLES**

**Table S1** Summary of significant SNP marker-trait associations for leaf chlorophyll fluorescence, shoot ionic, and grain quality traits

| **SNP** | **Associated traits** | **Chr** | **cM** | **P** | **Alleles** | **SNP R^2^** |
| --- | --- | --- | --- | --- | --- | --- |
| ***Leaf fluorescence traits*** |  |  |  |  |  |  |
| wsnp_Ex_rep_c66872_65273203 | ABS/RC | 1AL | 141.53 | 2.63E-07 | C/T | 3.2 |
| wsnp_RFL_Contig1984_1169021 | ABS/RC | 1DL | 91.53 | 4.95E-07 | A/G | 2.9 |
| BobWhite_c3871_210 | ABS/RC | 2DL | 80.41 | 2.66E-07 | C/T | 3.2 |
| BS00060391_51 | ABS/RC | 3AL | 111.62 | 7.74E-07 | G/A | 2.8 |
| TA003248_0911 | ABS/RC | 4BL | 68.45 | 3.74E-09 | C/A | 3.7 |
| BS00109052_51 | ABS/RC | 5A | 49.73 | 2.05E-07 | T/C | 3.3 |
| IACX5753 | ABS/RC | 6AL | 82.38 | 7.49E-11 | T/C | 4.8 |
| Kukri_rep_c107624_603 | ABS/RC | 6AL | 99.04 | 4.05E-10 | T/C | 4.4 |
| Ra_c106775_711 | ABS/RC | 6D | 82.14 | 2.46E-07 | C/T | 3.1 |
| BS00021955_51 | ABS/RC | 6AL | 81.96 | 1.89E-06 | T/C | 2.5 |
| IAAV1930 | DIo/RC | 1AL | 142.62 | 1.76E-06 | C/T | 6.2 |
| BS00021955_51 | DIo/RC | 5AL | 81.96 | 1.35E-13 | T/C | 7.3 |
| BS00003616_51 | DIo/RC | 6AL | 82.38 | 1.09E-25 | T/C | 14.9 |
| wsnp_Ex_c11348_18327861 | DIo/RC | 6AL | 85.07 | 2.74E-09 | A/G | 4.8 |
| Kukri_c15096_4206 | DIo/RC | 6AL | 99.04 | 2.02E-25 | T/C | 14.7 |
| RAC875_rep_c105906_124 | DIo/RC | 6BS | 23.32 | 4.76E-12 | A/G | 6.4 |
| Kukri_c9424_195 | DIo/RC | 6B | 46.96 | 4.86E-12 | A/G | 6.4 |
| wsnp_CV776265A_Ta_2_1 | DIo/RC | 6BL | 76.2 | 5.30E-12 | A/G | 6.4 |
| JD_c19177_1284 | DIo/RC | 7AL | 118.40 | 8.78E-09 | C/T |  |
| wsnp_Ex_c955_1827567 | ETo/RC | 1BL | 146.25 | 1.48E-09 | A/G | 5.4 |
| wsnp_Ex_c955_1827719 | ETo/RC | 1BL | 171.31 | 1.42E-08 | G/A | 4.8 |
| wsnp_Ex_rep_c66331_64502558 | ETo/RC | 3BS | 11.56 | 1.58E-08 | G/A | 4.7 |
| Kukri_c22602_791 | ETo/RC | 4AL | 154.3 | 4.50E-09 | C/T | 5 |
| BS00062617_51 | ETo/RC | 5BS | 5.7 | 1.19E-08 | C/T | 4.8 |
| wsnp_CAP8_c2589_1356390 | ETo/RC | 5D | 67.49 | 6.01E-09 | A/C | 4.9 |
| RAC875_c27986_1460 | F_v_ | 3BS | 4.54 | 2.44E-86 | A/G | 1.602 |
| Kukri_rep_c79597_513 | F_v_ | 4AS | 43.39 | 8.60E-237 | T/C | 9.246 |
| wsnp_BE591195A_Ta_1_1 | F_v_ | 4AS | 47.53 | 9.30E-156 | T/C | 3.902 |
| Kukri_rep_c103857_458 | F_v_ | 5A | 62.72 | 2.00E-127 | A/G | 2.825 |
| BS00062617_51 | F_v_ | 5BS | 5.7 | 4.50E-136 | C/T | 3.085 |
| wsnp_Ku_c1045_2115866 | F_v_ | 5BL | 143.55 | 8.79E-97 | T/C | 1.799 |
| wsnp_Ex_rep_c76495_73453891 | F_v_ | 6AL | 140.7036 | 3.15E-56 | C/T | 0.91461 |
| wsnp_CAP11_c651_429263 | F_v_ | 7AL | 127.75 | 1.50E-201 | G/A | 6.23 |
| wsnp_Ex_c1146_2200823 | F_v_ | 7AL | 131.11 | 2.60E-253 | A/G | 63.445 |
| RAC875_rep_c72959_187 | F_v_ | 7BL | 156.54 | 1.00E-189 | T/C | 21.959 |
| D_F1BEJMU02GB94Z_188 | F_m_/F_o_ | 2DS | 8.52 | 4.9332E-08 | G/A | 2.20 |
| Excalibur_c18417_285 | F_v_/F_m_ | 2BL | 98.53 | 5.35E-11 | T/C | 4 |
| CAP7_c3950_160 | F_v_/F_m_ | 7BL | 155.41 | 8.43E-08 | C/T | 3.4 |
| BS00021955_51 | F_v_/F_m_ | 5AL | 81.96 | 7.59E-05 | T/C |  |
| JD_c19177_1284 | F_v_/F_m_ | 7AL | 118.40 | 1.02E-05 | C/T |  |
| wsnp_Ku_c35386_44598937 | F_v_/F_o_ | 5A | 60.61 | 2.96E-09 | G/A | 2.5 |
| BS00003861_51 | F_v_/F_o_ | 6A | 48.09 | 6.80E-09 | T/C | 2.4 |
| CAP7_c3950_160 | F_v_/F_o_ | 7BL | 155.41 | 9.53E-13 | C/T | 3.6 |
| Tdurum_contig8448_363 | F_v_/F_o_ | 7BL | 164.24 | 3.49E-11 | A/C | 3.2 |
| Excalibur_rep_c110429_536 | F_v_/F_o_ | 7BL | 166.24 | 8.27E-12 | C/T | 3.5 |
| Kukri_c45404_121 | F_v_/F_o_ | 7BL | 171.11 | 4.18E-11 | C/T | 3.1 |
| GENE_4252_246 | TRo/RC | 3A | 77.57 | 5.85E-08 | G/A | 4.6 |
| CAP8_c1393_327 | TRo/RC | 3AL | 90.55 | 1.57E-09 | T/C | 5.8 |
| CAP7_rep_c12537_81 | TRo/RC | 3AL | 177.24 | 2.34E-08 | A/G | 5 |
| TA003248_0911 | TRo/RC | 4BL | 68.45 | 1.07E-05 | C/A | 4.10 |
| IACX11112 | TRo/RC | 7A | 74.25 | 9.29E-09 | G/C | 5.1 |
| CAP7_c3950_160 | PI_(ABS)_ | 7BL | 155.41 | 3.90E-05 | C/T | 4.50 |
| BS00009342_51 | PI_(ABS)_ | 4B | 75.65 | 8.50E-05 | A/G | 4.18 |
| BS00009342_51 | Psi_o | 4B | 75.65 | 3.47E-05 | A/G | 5.53 |
| RAC875_c68525_284 | PI_(ABS)_ | 6B | 76.79 | 1.50E-04 | G/A | 3.90 |
| Excalibur_c642_481 | Psi_o | 4B | 74.62 | 1.56E-04 | T/C | 4.66 |
| CAP7_c3950_160 | Psi_o | 7BL | 155.41 | 2.09E-04 | C/T | 3.62 |
| Tdurum_contig8448_363 | PI_(ABS)_ | 7BL | 164.24 | 3.04E-04 | A/C | 3.65 |
| BS00066456_51 | PI_(ABS)_ | 7BL | 163.91 | 3.29E-04 | A/G | 3.62 |
| Kukri_c5685_1066 | PI_(ABS)_ | 5BL | 115.69 | 3.58E-04 | C/T | 3.47 |
| ***Shoot ion contents after 25 day of salt treatment*** | | |  |  |  |  |
| RAC875_c36559_1994 | shoot K^+^/Na^+^ | 1BL | 85.57 | 8.69E-06 | G/A | 3.2 |
| Excalibur_c7971_1573 | shoot K^+^/Na^+^ | 2BL | 144.16 | 9.41E-07 | A/G | 4.1 |
| RFL_Contig3563_1130 | shoot K^+^/Na^+^ | 4BL | 68.45 | 2.20E-06 | C/T | 3.8 |
| Excalibur_c39621_358 | shoot K^+^/Na^+^ | 4AS | 43.39 | 1.18E-05 | G/A | 3.1 |
| Kukri_c59051_579 | shoot K^+^/Na^+^ | 5BL | 146.48 | 2.60E-06 | G/A | 3.6 |
| Kukri_rep_c107624_603 | shoot K^+^/Na^+^ | 6AL | 99.04 | 1.12E-5 | T/C | 2.4 |
| BS00099804_51 | shoot K^+^/Na^+^ | 7AL | 119.11 | 2.20E-06 | C/T | 3.5 |
| wsnp_Ex_c12117_19381493 | shoot Na^+^ | 1A | 70.1 | 2.69E-10 | C/A | 8 |
| Excalibur_rep_c69187_151 | shoot Na^+^ | 2BL | 99.73 | 2.19E-08 | A/G | 8.1 |
| BobWhite_c13455_112 | shoot Na^+^ | 2BL | 99.8 | 1.46E-08 | A/G | 8.3 |
| wsnp_Ku_c691_1430065 | shoot Na^+^ | 2BL | 99.87 | 1.06E-11 | C/T |  |
| Excalibur_c7971_1573 | shoot Na^+^ | 2BL | 144.16 | 3.39E-19 | A/G | 18.3 |
| BS00084096_51 | shoot Na^+^ | 5BL | 107.37 | 2.60E-08 | A/G | 7.9 |
| Tdurum_contig8171_1602 | shoot Na^+^ | 5BL | 140.17 | 3.17E-14 | T/C | 13.1 |
| BS00028082_51 | shoot Na^+^ | 5BL | 144.26 | 8.19E-14 | A/G |  |
| Kukri_c59051_579 | shoot Na^+^ | 5BL | 146.48 | 1.55E-15 | G/A | 13.3 |
| Ra_c45135_456 | shoot Na^+^ | 5BL | 144.12 | 1.89E-11 | G/T |  |
| Kukri_c21443_827 | shoot Na^+^ | 6AS | 28.46 | 1.01E-08 | C/T | 8.3 |
| BS00040124_51 | shoot Na^+^ | 6AL | 82.38 | 9.95E-09 | G/A | 8.1 |
| BS00003616_51 | shoot Na^+^ | 6AL | 82.38 | 6.33E-11 | T/C |  |
| IAAV5585 | shoot Na^+^ | 6AL | 99.04 | 1.19E-08 | G/T | 8.1 |
| Jagger_c1134_353 | shoot Na^+^ | 6AL | 140.87 | 7.25E-15 | A/G | 12 |
| RAC875_c25194_55 | shoot Na^+^ | 7AS | 35.31 | 4.27E-10 | G/A | 7.7 |
| ***Associated seed grain quality traits*** | |  |  |  |  |  |
| RAC875_c23168_480 | NDF | 1AL | 105.74 | 6.47E-11 | C/T | 4.6 |
| Excalibur_rep_c101324_1680 | NDF | 5AS | 26.51 | 9.24E-10 | C/T | 4.1 |
| wsnp_Ex_rep_c68117_66883366 | NDF | 5AS | 43.27 | 1.00E-09 | G/A | 4.1 |
| wsnp_Ex_rep_c68269_67060931 | NDF | 5AS | 43.44 | 2.21E-10 | G/T | 4.5 |
| Kukri_c15761_1634 | NDF | 6B | 71.76 | 7.40E-10 | C/T | 4.1 |
| wsnp_Ex_c18499_27344859 | GPC | 1AL | 137.12 | 7.84E-13 | C/T | 4.6 |
| BS00022824_51 | GPC | 1AL | 137.69 | 1.84E-12 | T/C | 4.4 |
| BS00011521_51 | GPC | 1AL | 139.74 | 6.73E-12 | A/G | 4.1 |
| wsnp_Ex_c742_1458743 | GPC | 3AL | 81.82 | 1.43E-11 | G/T | 4.2 |
| wsnp_Ex_c18499_27344859 | **GSC** | 1AL | 137.12 | 5.77E-05 | C/T | 0.13 |
| BS00023114_51 | **GSC** | 1AL | 137.20 | 6.83E-05 | C/T | 0.13 |
| Excalibur_c60683_908 | GPC | 5B | 49.01 | 7.19E-11 | T/C | 3.7 |
| RFL_Contig4251_851 | GPC | 6B | 71.9 | 3.04E-12 | A/G | 4.4 |
| Kukri_c15761_1634 | **CFC** | 6B | 71.76 | 1.75E-09 | C/T | 0.12 |
| IAAV4238 | CFC | 1AL | 144.94 | 6.18E-14 | G/A | 5.6 |
| BS00110480_51 | CFC | 1B | 68.04 | 3.27E-12 | G/A | 5.2 |
| BobWhite_c2058_367 | CFC | 2AL | 119.93 | 6.65E-10 | C/T | 4 |
| BobWhite_c13455_112 | CFC | 2BL | 99.8 | 8.11E-09 | A/G | 3.5 |
| RAC875_c25656_289 | CFC | 2DS | 8.52 | 5.85E-09 | C/T | 3.5 |
| Excalibur_c8768_1163 | CFC | 2DL | 99.19 | 1.44E-10 | A/G | 4.2 |
| Excalibur_c9619_1136 | CFC | 2DS | 100.58 | 2.02E-09 | G/A | 3.8 |
| RFL_Contig2862_1219 | CFC | 2DS | 105.13 | 3.39E-11 | A/G | 4.6 |
| RAC875_c19099_434 | CFC | 5BL | 68.93 | 1.40E-12 | C/T | 5.2 |

**NDF**, neutral detergent fiber; GPC, protein content; **CFC**, crude fiber

**Table S2** Gene content of the associated genetic interval in the scaffold41600 (~13 Mb) as revealed in the IWGSC database in their natural order

| **S/N** | **Gene ID** | **Quality-Code** | **Gene annotation** | **Abundances (%)** | |
| --- | --- | --- | --- | --- | --- |
|  |  |  |  | **Altay2000** | **Bobur** |
| 1 | TraesCS4B01G250700.1 | --* | galacturonic acid kinase | -10.62 | -44.34 |
| 2 | TraesCS4B01G250800.1 | NA | NA | 0 | 0 |
| 3 | TraesCS4B01G250800.2 | *** | Pentatricopeptide repeat-containing protein | -46.28 | -34.66 |
| 4 | TraesCS4B01G250800.3 | NA | NA | 0 | 0 |
| 5 | TraesCS4B01G250900.1 | *-* | Remorin family protein | -100 | 0 |
| 6 | TraesCS4B01G250900.2 | NA | NA | 0 | 0 |
| 7 | TraesCS4B01G251000.1 | *-* | Plant calmodulin-binding-like protein | 0 | 0 |
| 8 | TraesCS4B01G251100.1 | *** | Glucan endo-1,3-beta-glucosidase 3 | 164.01 | 66.98 |
| 9 | TraesCS4B01G251200.1 | --* | E3 ubiquitin-protein ligase | -0.69 | -2.11 |
| 10 | TraesCS4B01G251300.1 | *** | G-patch domain containing protein, expressed | -0.69 | 11.32 |
| 11 | TraesCS4B01G251400.1 | *-* | DNA-directed RNA polymerase subunit beta | 86.20 | 87.85 |
| 12 | TraesCS4B01G251500.1 | *** | B3 domain-containing protein | 0 | 0 |
| 13 | TraesCS4B01G251600.1 | NA | NA | 0 | 0 |
| 14 | TraesCS4B01G251600.2 | *** | Poly(A) RNA polymerase cid14 | 102.59 | 63.19 |
| 15 | TraesCS4B01G251700.1 | *** | Acetyl-CoA decarbonylase/synthase complex subunit alpha 1 | -25.02 | 1.62 |
| 16 | TraesCS4B01G251800.1 | NA | NA | 0 | 0 |
| 17 | TraesCS4B01G251800.2 | *-* | Nodulin-related protein 1, putative | 101.44 | 41.22 |
| 18 | TraesCS4B01G251900.1 | *** | DUF538 family protein (Protein of unknown function, DUF538) | 30.34 | 173.24 |
| 19 | TraesCS4B01G252000.1 | *-* | WEAK movement UNDER BLUE LIGHT-like protein | 0 | 0 |
| 20 | TraesCS4B01G252100.1 | NA | NA | 0 | 0 |
| 21 | TraesCS4B01G252100.2 | *** | Beta-xylosidase, putative | -0.69 | 0 |
| 22 | TraesCS4B01G252200.1 | *** | Cysteine proteinase inhibitor | 0 | 0 |
| 23 | TraesCS4B01G252300.1 | *** | Cysteine proteinase inhibitor | 0 | 0 |
| 24 | TraesCS4B01G252400.1 | *** | Methionine--tRNA ligase | -14.88 | -26.95 |
| 25 | TraesCS4B01G252400.2 | NA | NA | 0 | 0 |
| 26 | TraesCS4B01G252400.3 | NA | NA | 0 | 0 |
| 27 | TraesCS4B01G252500.1 | *** | S-acyltransferase | -100 | -16.51 |
| 28 | TraesCS4B01G252600.1 | *** | Protein transport protein GOT1 | -100 | -100 |
| 29 | TraesCS4B01G252700.1 | NA | NA | 0 | 0 |
| 30 | TraesCS4B01G252800.1 | --* | GDP-L-galactose phosphorylase 1 | 64.07 | -35.99 |
| 31 | TraesCS4B01G252900.1 | *-* | RAN GTPase-activating protein 2 | 12.55 | -25.07 |
| 32 | TraesCS4B01G253000.1 | *** | Mannose-1-phosphate guanyltransferase | 14.96 | -10.03 |
| 33 | TraesCS4B01G253100.1 | *** | Cyclin | 197.92 | -55.80 |
| 34 | TraesCS4B01G253200.1 | *** | Multidrug resistance protein ABC transporter family protein | 45.50 | -32.16 |
| 35 | TraesCS4B01G253300.1 | NA | NA | 0 | 0 |
| 36 | TraesCS4B01G253300.2 | *** | Alpha-L-fucosidase 2 | 48.96 | -13.20 |
| 37 | TraesCS4B01G253300.3 | NA | NA | 0 | 0 |
| 38 | TraesCS4B01G253400.1 | --* | protein phosphatase 2A 55 kDa regulatory subunit B alpha isoform | 0 | 0 |
| 39 | TraesCS4B01G253500.1 | *** | Alpha-L-fucosidase 2 | 48.96 | 55.05 |
| 40 | TraesCS4B01G253600.1 | NA | NA | 0 | 0 |
| 41 | TraesCS4B01G253700.1 | *** | Dirigent protein | 0 | 0 |
| 42 | TraesCS4B01G253800.1 | --* | cytochrome P450, family 71, subfamily B, polypeptide 12 | 48.96 | -100.00 |
| 43 | TraesCS4B01G253900.1 | *** | 2-oxoglutarate-dependent dioxygenase-related family protein | 0 | -100 |
| 44 | TraesCS4B01G254000.1 | *-* | Cyclin | 0 | 0 |
| 45 | TraesCS4B01G254100.1 | *** | Cysteine/Histidine-rich C1 domain family protein | 48.96 | 150.47 |
| 46 | TraesCS4B01G254200.1 | *** | Pentatricopeptide repeat-containing protein, putative | 22.67 | 7.34 |
| 47 | TraesCS4B01G254300.1 | *** | Metal transporter (OPAQUE1) | 363.43 | -73.49 |
| 48 | TraesCS4B01G254400.1 | *** | WD-repeat protein, putative | 18.49 | -29.61 |
| 49 | TraesCS4B01G254400.2 | NA | NA | 0 | 0 |
| 50 | TraesCS4B01G254400.3 | NA | NA | 0 | 0 |
| 51 | TraesCS4B01G254500.1 | *** | Tetratricopeptide repeat protein 1 | 0 | 0 |
| 52 | TraesCS4B01G254600.1 | --* | Glutathione S-transferase T3 | 0 | 0 |
| 53 | TraesCS4B01G254700.1 | *** | Phosphatase 2C family protein | 11.72 | 138.54 |
| 54 | TraesCS4B01G254800.1 | *** | Protein kinase | 33.63 | 10.14 |
| 55 | TraesCS4B01G254800.2 | NA | NA | 0 | 0 |
| 56 | TraesCS4B01G254900.1 | *** | 60S ribosomal protein L51, mitochondrial | 4.82 | 16.29 |
| 57 | TraesCS4B01G255000.1 | --* | Syntaxin protein | 68.39 | 32.52 |
| 58 | TraesCS4B01G255100.1 | *** | F-box protein | -25.52 | 7.34 |
| 59 | TraesCS4B01G255200.1 | *** | Dirigent protein | 0 | 0 |
| 60 | TraesCS4B01G255300.1 | *** | F-box protein | -44.14 | 43.13 |
| 61 | TraesCS4B01G255400.1 | *-* | RING/U-box superfamily protein | 23.05 | 17.02 |

**Table S3** Gene content of the associated genetic interval in the *scaffold126294.1* *(*~12 Mb) as revealed in the IWGSC database in their natural order

| **S/N** | **Gene ID** | **Quality-Code** | **Gene annotation** | **Transcript abundance (%)** | |
| --- | --- | --- | --- | --- | --- |
|  |  |  |  | **Altay2000** | **Bobur** |
| 1 | TraesCS6A01G331300.1 | NA | NA | -5.72 | -61.47 |
| 2 | TraesCS6A01G331300.2 | *** | PF00201: UDP-glucoronosyl and UDP-glucosyl transferase | -5.72 | -61.47 |
| 3 | TraesCS6A01G331400.1 | *** | PF14226: non-haem dioxygenase in morphine synthesis N-terminal; PF03171: 2OG-Fe(II) oxygenase superfamily | 0.00 | 0.00 |
| 4 | TraesCS6A01G331500.1 | *** | PF00134: Cyclin, N-terminal domain | 70.94 | 23.47 |
| 5 | TraesCS6A01G331600.1 | *** | PF00083: Sugar (and other) transporter | 197.92 | -16.51 |
| 6 | TraesCS6A01G331700.1 | *-* | PF03692: Putative zinc- or iron-chelating domain | -33.80 | 122.64 |
| 7 | TraesCS6A01G331800.1 | *** | PF04305: Protein of unknown function (DUF455) | -31.25 | -22.93 |
| 8 | TraesCS6A01G331900.1 | *** | NA | 45.41 | 39.80 |
| 9 | TraesCS6A01G332000.1 | *** | PF01641: SelR domain | -35.47 | -24.92 |
| 10 | TraesCS6A01G332100.1 | *** | PF01764: Lipase (class 3) | 0 | -100.00 |
| 11 | TraesCS6A01G332200.1 | --* | NA | 0 | 0 |
| 12 | TraesCS6A01G332300.1 | *** | PF00967: Barwin family | 0 | 0 |
| 13 | TraesCS6A01G332400.1 | --* | PF00967: Barwin family | 0 | 0 |
| 14 | TraesCS6A01G332500.1 | *** | PF00967: Barwin family | 0 | 0 |
| 15 | TraesCS6A01G332600.1 | *** | PF00967: Barwin family | 0 | 0 |
| 16 | TraesCS6A01G332700.1 | NA | NA | 0 | 0 |
| 17 | TraesCS6A01G332800.1 | *** | PF09766: Fms-interacting protein | 22.15 | 5.85 |
| 18 | TraesCS6A01G332900.1 | *** | NA | 0 | 0 |
| 19 | TraesCS6A01G333000.1 | *** | PF00717: Peptidase S24-like | 0 | 0 |
| 20 | TraesCS6A01G333000.2 | NA | NA | 0 | 0 |
| 21 | TraesCS6A01G333100.1 | *** | PF08314: Secretory pathway protein Sec39 | 39.93 | -36.45 |
| 22 | TraesCS6A01G333200.1 | *** | PF03763: Remorin, C-terminal region | 0 | 0 |
| 23 | TraesCS6A01G333300.1 | *** | PF00150: Cellulase (glycosyl hydrolase family 5) | 0 | 0 |
| 24 | TraesCS6A01G333400.1 | *** | PF00150: Cellulase (glycosyl hydrolase family 5) | -55.86 | -22.81 |
| 25 | TraesCS6A01G333500.1 | *** | PF00150: Cellulase (glycosyl hydrolase family 5) | 0 | 0 |
| 26 | TraesCS6A01G333600.1 | NA | NA | 372.36 | 64.62 |
| 27 | TraesCS6A01G333600.2 | *** | PF00170: bZIP transcription factor | 372.36 | 64.62 |
| 28 | TraesCS6A01G333700.1 | *** | PF07939: Protein of unknown function (DUF1685) | 35.72 | -3.39 |
| 29 | TraesCS6A01G333800.1 | *-* | PF11523: Protein of unknown function (DUF3223) | -11.83 | 7.63 |
| 30 | TraesCS6A01G333900.1 | *** | PF03460: Nitrite/Sulfite reductase ferredoxin-like half domain; PF01077: Nitrite and sulphite reductase 4Fe-4S domain | -61.72 | -44.42 |
| 31 | TraesCS6A01G334000.1 | *** | PF02036: SCP-2 sterol transfer family | 152.09 | 51.91 |
| 32 | TraesCS6A01G334100.1 | *** | PF00128: Alpha amylase, catalytic domain; PF07821: Alpha-amylase C-terminal beta-sheet domain | 0 | 0 |
| 33 | TraesCS6A01G334200.1 | *** | PF00128: Alpha amylase, catalytic domain; PF07821: Alpha-amylase C-terminal beta-sheet domain | 0 | 0 |
| 34 | TraesCS6A01G334300.1 | *** | PF02403: Seryl-tRNA synthetase N-terminal domain; PF00587: tRNA synthetase class II core domain (G, H, P, S and T) | -3.10 | -39.96 |
| 35 | TraesCS6A01G334400.1 | --* | NA | -25.52 | -16.51 |
| 36 | TraesCS6A01G334500.1 | *** | PF00069: Protein kinase domain | 0 | 0 |
| 37 | TraesCS6A01G334600.1 | *** | PF00717: Peptidase S24-like | 0 | 0 |
| 38 | TraesCS6A01G334700.1 | NA | NA | 91.52 | 66.98 |
| 39 | TraesCS6A01G334700.2 | *** | PF13532: 2OG-Fe(II) oxygenase superfamily | 91.52 | 66.98 |
| 40 | TraesCS6A01G334800.1 | *** | PF06351: Allene oxide cyclase | 522.76 | 3.20 |
| 41 | TraesCS6A01G334900.1 | NA | NA | 123.44 | -18.09 |
| 42 | TraesCS6A01G334900.2 | NA | NA | 123.44 | -18.09 |
| 43 | TraesCS6A01G334900.3 | *** | PF00225: Kinesin motor domain | 123.44 | -18.09 |
| 44 | TraesCS6A01G335000.1 | *** | NA | 0 | 0 |
| 45 | TraesCS6A01G335100.1 | *** | NA | -42.71 | 5.46 |
| 46 | TraesCS6A01G335200.1 | -** | NA | 227.71 | 267.36 |
| 47 | TraesCS6A01G335300.1 | *-* | PF05605: Drought induced 19 protein (Di19), zinc-binding | 0 | 0 |
| 48 | TraesCS6A01G335400.1 | *** | PF02045: CCAAT-binding transcription factor (CBF-B/NF-YA) subunit B | -20.55 | -47.05 |
| 49 | TraesCS6A01G335500.1 | *** | PF00010: Helix-loop-helix DNA-binding domain | 0 | 0 |
| 50 | TraesCS6A01G335600.1 | --* | NA | 104.82 | 59.39 |
| 51 | TraesCS6A01G335700.1 | *** | PF04057: Replication factor-A protein 1, N-terminal domain; PF01336: OB-fold nucleic acid binding domain; PF16900: Replication protein A OB domain; PF08646: Replication factor-A C terminal domain | 11.72 | 25.24 |
| 52 | TraesCS6A01G335800.1 | *** | PF04110: Ubiquitin-like autophagy protein Apg12 | 30.34 | 32.60 |
| 53 | TraesCS6A01G335800.2 | NA | NA | 30.34 | 32.60 |
| 54 | TraesCS6A01G335900.1 | *** | PF08880: QLQ; PF08879: WRC | 0 | 0 |
| 55 | TraesCS6A01G336000.1 | *** | PF03456: uDENN domain; PF02141: DENN (AEX-3) domain | -30.59 | -7.77 |
| 56 | TraesCS6A01G336000.2 | NA | NA | -30.59 | -7.77 |
| 57 | TraesCS6A01G336000.3 | NA | NA | -30.59 | -7.77 |
| 58 | TraesCS6A01G336000.4 | NA | NA | -30.59 | -7.77 |
| 59 | TraesCS6A01G336100.1 | *** | PF02458: Transferase family | 0 | 0 |
| 60 | TraesCS6A01G336200.1 | NA | NA | 0 | 0 |
| 61 | TraesCS6A01G336300.1 | *** | PF08263: Leucine rich repeat N-terminal domain; PF13855: Leucine rich repeat; PF13516: Leucine Rich repeat; PF00069: Protein kinase domain | 495.84 | -72.17 |
| 62 | TraesCS6A01G336400.1 | --* | NA | 0 | 0 |
| 63 | TraesCS6A01G336500.1 | *** | PF00063: Myosin head (motor domain); PF00612: IQ calmodulin-binding motif; PF01843: DIL domain | 48.96 | 66.98 |
| 64 | TraesCS6A01G336600.1 | *** | PF07714: Protein tyrosine kinase | 3524.72 | 734.90 |
| 65 | TraesCS6A01G336700.1 | *** | PF07893: Protein of unknown function (DUF1668) | 197.92 | 567.92 |
| 66 | TraesCS6A01G336800.1 | *** | PF00133: tRNA synthetases class I (I, L, M and V); PF08264: Anticodon-binding domain of tRNA | -22.83 | -12.59 |
| 67 | TraesCS6A01G336900.1 | *** | PF14802: TMEM192 family | 36.88 | -40.80 |
| 68 | TraesCS6A01G336900.2 | NA | NA | 36.88 | -40.80 |
| 69 | TraesCS6A01G337000.1 | NA | NA | -11.85 | -9.68 |
| 70 | TraesCS6A01G337000.2 | NA | NA | -11.85 | -9.68 |
| 71 | TraesCS6A01G337000.3 | NA | NA | -11.85 | -9.68 |
| 72 | TraesCS6A01G337000.4 | *** | PF13041: PPR repeat family; PF17177: Pentacotripeptide-repeat region of PROPR; PF01535: PPR repeat | -11.85 | -9.68 |
| 73 | TraesCS6A01G337000.5 | NA | NA | -11.85 | -9.68 |
| 74 | TraesCS6A01G337000.6 | NA | NA | -11.85 | -9.68 |
| 75 | TraesCS6A01G337100.1 | NA | NA | 16.29 | 74.74 |
| 76 | TraesCS6A01G337100.2 | NA | NA | 16.29 | 74.74 |
| 77 | TraesCS6A01G337100.3 | NA | NA | 16.29 | 74.74 |
| 78 | TraesCS6A01G337100.4 | *** | PF00012: Hsp70 protein | 16.29 | 74.74 |
| 79 | TraesCS6A01G337200.1 | *** | PF16035: Chalcone isomerase like | -18.75 | -88.61 |
| 80 | TraesCS6A01G337300.1 | *** | PF00759: Glycosyl hydrolase family 9 | 2.14 | 53.07 |
| 81 | TraesCS6A01G337400.1 | --* | PF14364: Domain of unknown function (DUF4408) | -25.52 | 150.47 |
| 82 | TraesCS6A01G337500.1 | --* | NA | 0 | 0 |
| 83 | TraesCS6A01G337600.1 | *** | PF00722: Glycosyl hydrolases family 16; PF06955: Xyloglucan endo-transglycosylase (XET) C-terminus | 0 | 0 |
| 84 | TraesCS6A01G337700.1 | *** | NA | 48.96 | 192.22 |
| 85 | TraesCS6A01G337800.1 | *** | PF05922: Peptidase inhibitor I9; PF00082: Subtilase family; PF02225: PA domain | 0 | 0 |
| 86 | TraesCS6A01G337900.1 | *** | PF05922: Peptidase inhibitor I9; PF00082: Subtilase family; PF02225: PA domain | 0 | 0 |
| 87 | TraesCS6A01G338000.1 | *-* | PF05687: BES1/BZR1 plant transcription factor, N-terminal | 0 | 0 |
| 88 | TraesCS6A01G338100.1 | NA | NA | 0 | 0 |
| 89 | TraesCS6A01G338200.1 | *-* | PF00069: Protein kinase domain | 0 | 0 |
| 90 | TraesCS6A01G338200.2 | NA | NA | 0 | 0 |
| 91 | TraesCS6A01G338300.1 | *** | PF07714: Protein tyrosine kinase; PF04564: U-box domain | 0 | 0 |
| 92 | TraesCS6A01G338400.1 | *-* | PF00319: SRF-type transcription factor (DNA-binding and dimerisation domain) | 0 | 0 |
| 93 | TraesCS6A01G338500.1 | *** | PF05922: Peptidase inhibitor I9 | 0 | 0 |
| 94 | TraesCS6A01G338600.1 | *** | PF00155: Aminotransferase class I and II | 167.52 | -4.99 |
| 95 | TraesCS6A01G338600.2 | NA | NA | 167.52 | -4.99 |
| 96 | TraesCS6A01G338700.1 | --* | NA | 78.75 | 39.15 |
| 97 | TraesCS6A01G338800.1 | *** | PF00145: C-5 cytosine-specific DNA methylase | 48.96 | 0.00 |
| 98 | TraesCS6A01G338800.2 | NA | NA | 48.96 | 0.00 |
| 99 | TraesCS6A01G338900.1 | *** | PF01535: PPR repeat; PF13041: PPR repeat family | 98.61 | 0.00 |
| 100 | TraesCS6A01G339000.1 | *-* | NA | 0 | 0 |
| 101 | TraesCS6A01G339100.1 | *** | PF05922: Peptidase inhibitor I9; PF00082: Subtilase family; PF02225: PA domain | 0 | 0 |
| 102 | TraesCS6A01G339200.1 | *** | PF05922: Peptidase inhibitor I9; PF00082: Subtilase family; PF02225: PA domain | 0 | 0 |
| 103 | TraesCS6A01G339300.1 | *** | PF00657: GDSL-like Lipase/Acylhydrolase | 0 | 0 |
| 104 | TraesCS6A01G339400.1 | *** | PF05922: Peptidase inhibitor I9; PF00082: Subtilase family; PF02225: PA domain | 28.83 | 130.99 |
| 105 | TraesCS6A01G339500.1 | NA | NA | 98.61 | 16.89 |
| 106 | TraesCS6A01G339500.2 | *** | NA | 98.61 | 16.89 |
| 107 | TraesCS6A01G339600.1 | *** | PF00445: Ribonuclease T2 family | 183.60 | 98.29 |
| 108 | TraesCS6A01G339700.1 | *** | NA | -100.00 | 0 |
| 109 | TraesCS6A01G339800.1 | *** | PF03893: Lipase 3 N-terminal region; PF01764: Lipase (class 3) | -100.00 | -100.00 |
| 110 | TraesCS6A01G339900.1 | *-* | PF00270: DEAD/DEAH box helicase; PF00271: Helicase conserved C-terminal domain; PF16124: RecQ zinc-binding | -40.42 | -30.42 |
| 111 | TraesCS6A01G340000.1 | *** | PF00295: Glycosyl hydrolases family 28 | 644.80 | 25.24 |
| 112 | TraesCS6A01G340100.1 | *-* | PF00847: AP2 domain | 0 | 0 |
| 113 | TraesCS6A01G340200.1 | *-* | PF00795: Carbon-nitrogen hydrolase | -100.00 | 0 |
| 114 | TraesCS6A01G340300.1 | NA | NA | -86.76 | -76.15 |
| 115 | TraesCS6A01G340300.2 | *-* | PF00166: Chaperonin 10 Kd subunit | -86.76 | -76.15 |
| 116 | TraesCS6A01G340300.3 | NA | NA | -86.76 | -76.15 |
| 117 | TraesCS6A01G340400.1 | *** | PF01663: Type I phosphodiesterase / nucleotide pyrophosphatase | 115.17 | -43.52 |
| 118 | TraesCS6A01G340500.1 | NA | NA | -30.15 | -36.09 |
| 119 | TraesCS6A01G340500.2 | *** | NA | -30.15 | -36.09 |
| 120 | TraesCS6A01G340500.3 | NA | NA | -30.15 | -36.09 |
| 121 | TraesCS6A01G340600.1 | *-* | PF00642: Zinc finger C-x8-C-x5-C-x3-H type (and similar) | 31.77 | 34.09 |
| 122 | TraesCS6A01G340700.1 | --* | PF00646: F-box domain | 73.79 | 4.36 |
| 123 | TraesCS6A01G340800.1 | NA | NA | 0 | 0 |
| 124 | TraesCS6A01G340900.1 | NA | NA | 0 | -28.44 |
| 125 | TraesCS6A01G341000.1 | --* | PF00646: F-box domain | 197.92 | -100.00 |
| 126 | TraesCS6A01G341100.1 | NA | NA | 11.72 | 0 |
| 127 | TraesCS6A01G341200.1 | --* | NA | 0 | 0 |
| 128 | TraesCS6A01G341300.1 | *** | PF03478: Protein of unknown function (DUF295) | -100.00 | 0 |
| 129 | TraesCS6A01G341400.1 | *** | PF08392: FAE1/Type III polyketide synthase-like protein; PF08541: 3-Oxoacyl-[acyl-carrier-protein (ACP)] synthase III C terminal | 0 | 0 |
| 130 | TraesCS6A01G341500.1 | --* | NA | 91.52 | 0 |
| 131 | TraesCS6A01G341600.1 | --* | NA | 0 | -100.00 |
| 132 | TraesCS6A01G341600.2 | NA | NA | 0 | -100.00 |
| 133 | TraesCS6A01G341700.1 | *** | PF08161: NUC173 domain | 29.43 | -14.01 |
| 134 | TraesCS6A01G341800.1 | *** | PF03080: Domain of unknown function (DUF239) | 0 | -30.42 |
| 135 | TraesCS6A01G341900.1 | *** | PF00226: DnaJ domain | 84.71 | -6.07 |
| 136 | TraesCS6A01G342000.1 | *** | PF13947: Wall-associated receptor kinase galacturonan-binding; PF07645: Calcium-binding EGF domain; PF00069: Protein kinase domain | 0 | 0 |
| 137 | TraesCS6A01G342100.1 | --* | NA | -100.00 | 0 |
| 138 | TraesCS6A01G342200.1 | *** | PF00012: Hsp70 protein | 0 | 0 |
| 139 | TraesCS6A01G342300.1 | --* | PF06839: GRF zinc finger | 0 | 0 |
| 140 | TraesCS6A01G342400.1 | *** | PF00012: Hsp70 protein | -50.35 | 0 |
| 141 | TraesCS6A01G342500.1 | *** | PF00012: Hsp70 protein | 0 | 0 |
